# Online Self-Administered Cognitive Testing Using the Amsterdam Cognition Scan: Establishing Psychometric Properties and Normative Data

**DOI:** 10.2196/jmir.9298

**Published:** 2018-05-30

**Authors:** Heleen EM Feenstra, Ivar E Vermeulen, Jaap MJ Murre, Sanne B Schagen

**Affiliations:** ^1^ Department of Psychosocial Research and Epidemiology Netherlands Cancer Institute Amsterdam Netherlands; ^2^ Department of Psychology University of Amsterdam Amsterdam Netherlands; ^3^ Department of Communication Science VU University Amsterdam Amsterdam Netherlands

**Keywords:** cognition, neuropsychological tests, self-assessment, internet, reproducibility of results, reference standards

## Abstract

**Background:**

Online tests enable efficient self-administered assessments and consequently facilitate large-scale data collection for many fields of research. The Amsterdam Cognition Scan is a new online neuropsychological test battery that measures a broad variety of cognitive functions.

**Objective:**

The aims of this study were to evaluate the psychometric properties of the Amsterdam Cognition Scan and to establish regression-based normative data.

**Methods:**

The Amsterdam Cognition Scan was self-administrated twice from home—with an interval of 6 weeks—by 248 healthy Dutch-speaking adults aged 18 to 81 years.

**Results:**

Test-retest reliability was moderate to high and comparable with that of equivalent traditional tests (intraclass correlation coefficients: .45 to .80; .83 for the Amsterdam Cognition Scan total score). Multiple regression analyses indicated that (1) participants’ age negatively influenced all (12) cognitive measures, (2) gender was associated with performance on six measures, and (3) education level was positively associated with performance on four measures. In addition, we observed influences of tested computer skills and of self-reported amount of computer use on cognitive performance. Demographic characteristics that proved to influence Amsterdam Cognition Scan test performance were included in regression-based predictive formulas to establish demographically adjusted normative data.

**Conclusions:**

Initial results from a healthy adult sample indicate that the Amsterdam Cognition Scan has high usability and can give reliable measures of various generic cognitive ability areas. For future use, the influence of computer skills and experience should be further studied, and for repeated measurements, computer configuration should be consistent. The reported normative data allow for initial interpretation of Amsterdam Cognition Scan performances.

## Introduction

### Online Cognitive Assessments

Following the rise of computerized cognitive testing over the past decades [1,2], online cognitive testing is now also increasingly applied in both research and clinical practice [3]. Online testing shares its main advantages with computerized testing: standardization and precise (multiple) response measurements [4-6] but adds to that the advantages associated with self-administered testing: flexibility (in time and location) and cost-efficiency [1,7-11]. Importantly, this allows online assessments to take place from home. Furthermore, central management of online test platforms allows for continuous software updates and opens ways for gathering normative data during testing. In sum, online testing may greatly facilitate large-scale cognitive data collection, which is needed in many fields of research [12-19]. One of these fields is oncology. Many cancer patients develop cognitive problems during the course of their disease [20]. With the growing community of cancer survivors and the increasingly chronic nature of many common cancers, the management of symptoms related to the disease and its treatments has become an important part of long-term survivorship care [21].

### Development of the Amsterdam Cognition Scan

To advance our understanding of cognitive decline in cancer patients, we developed a self-administered online neuropsychological test battery: the Amsterdam Cognition Scan (ACS). The goal of the ACS is to measure broad cognitive functioning for research purposes. Although this new test battery was designed for the oncology setting, it measures various cognitive abilities and is therefore equally suitable for cognitive studies in other settings. The ACS measures attention, information processing, learning and memory, executive functioning, and psychomotor speed. Psychometric properties have been studied in Dutch adult noncentral nervous system cancer patients. Overall, adequate test-retest reliability was observed (n=96; 59% [57/96] female; mean age 51.8 years, SD 11.9; 57% [54/96] high education level), with correlations comparable with those of equivalent traditional tests (*r* / *ρ*=.29-.79) [22]. A second study (n=201; 55.7% [112/201] female; mean age 53.5 years, SD 12.3; 61.2% [123/201] high education level) showed concurrent validity, ie, consistency with scores from equivalent traditional tests, to be medium to large (*r* / *ρ*=.42-.70; total score *r*=.78), except for a visuospatial memory test (*ρ*=.36) [22]. Correlations were affected—as expected—by design differences between online tests and their offline counterparts. Furthermore, usability proved to be high as almost all participants could successfully complete the test battery from home—unsupervised and without technical problems. Most of these participants (90%) indicated to prefer online home assessments over online or traditional assessments from the hospital.

### Objectives

To further facilitate use of the ACS, we collected reference data in a sample of 248 healthy adults. This enables indicating sensitivity to demographic characteristics, which is relevant as age, education, and—to a lesser extent—gender are often found to be associated with (various types of) cognitive performance [23,24]. Even though every new test requires representative normative data [25,26], online neuropsychological tests that are currently available often lack normative data or use data from offline assessments [1,27].

In sum, the aims of this study were to evaluate the psychometric properties of the ACS in a healthy sample and to establish regression-based demographically corrected normative data.

## Methods

### Participants

Reference data were collected from 248 healthy Dutch-speaking adults. All participants were recruited (October 2013-November 2014) via cancer patients of the Netherlands Cancer Institute who were participating in the ACS validation study [22]. First, patients of the validation study provided contact details of friends or family members suitable for participation in the study. Subsequently, the research team sent out invitation letters and contacted invitees by telephone 2 weeks later. Those who were interested in participation were asked several questions to verify eligibility. All participants were required to have sufficient proficiency of the Dutch language, basic computer skills (ie, being able to operate the mouse and send emails independently), and access to a computer with an Internet connection. Exclusion criteria were history of cancer and self-reported neurological or psychiatric conditions that could influence cognitive functioning (eg, schizophrenia, psychosis, clinical depression, substance dependence, or brain pathology). Because one of the seven neuropsychological tests—Place the Beads—was still under development during data collection, data for this test were obtained later in a different sample of 421 healthy Dutch adults that were recruited through an online respondent panel using similar inclusion and exclusion criteria as for the main reference sample.

### Procedure

The study was approved by the review board of the Netherlands Cancer Institute conform ethical guidelines for human experimentation stated in the Declaration of Helsinki. Before the start of the assessments, informed consent was obtained from all participants. Assessments were completed in an unmonitored setting, either from home or from other private locations. Participants were instructed to find a quiet environment without distractions and to complete the test in one sitting. First, the ACS was administered: seven neuropsychological tests and two questionnaires—the Hospital Anxiety and Depression Scale (HADS) [28] on symptoms of depression and anxiety and the Multidimensional Fatigue Inventory-20 (MFI-20) [29] on fatigue—were presented. The complete battery—including two fixed, standardized breaks of 2 min each—took about 1 hour (on average 56 min for the first assessment) to complete. The ACS was followed by an online debriefing on test conditions during the assessment (eg, disruptions and technical issues). Around 6 weeks later (median=45 days; SD=13.8), the ACS was repeated using the same order and versions of neuropsychological tests.

For the Place the Beads reference sample, subscribers to an online respondent panel were stratified to match demographic characteristics of the main reference sample and subsequently invited by email. Participants from this sample received similar instructions but were presented with the Place the Beads test only. Both participants and nonparticipants received a second invitation, either as a reminder or as an invitation to repeat the test after 6 to 8 weeks (median=79 days; SD=29.5).

### Computer Skills and Neuropsychological Assessment

**Figure 1:**
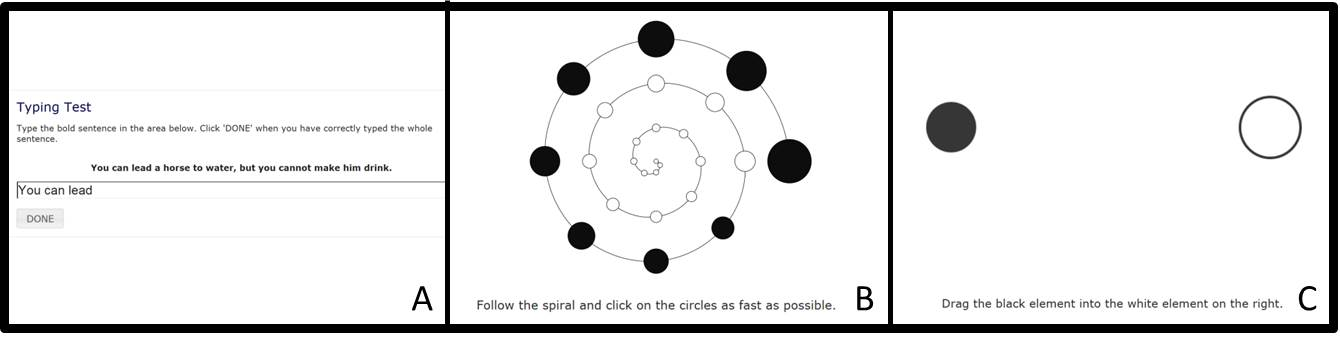
Computer skill test from the Amsterdam Cognition Scan.

Before the start of the first neuropsychological test, computer skills were assessed via tests of keyboard type skills (Type Skills), mouse click skills (Click Skills), and mouse drag skills (Drag Skills) that were newly developed by the research team (see Multimedia Appendix 1 for a description of these tests and Figure 1 for screenshots of these tests). To create a compound score that reflects overall computer skills, standardized scores for these three tests were averaged, such that higher scores indicate better overall computer skills [22].

**Figure 2:**
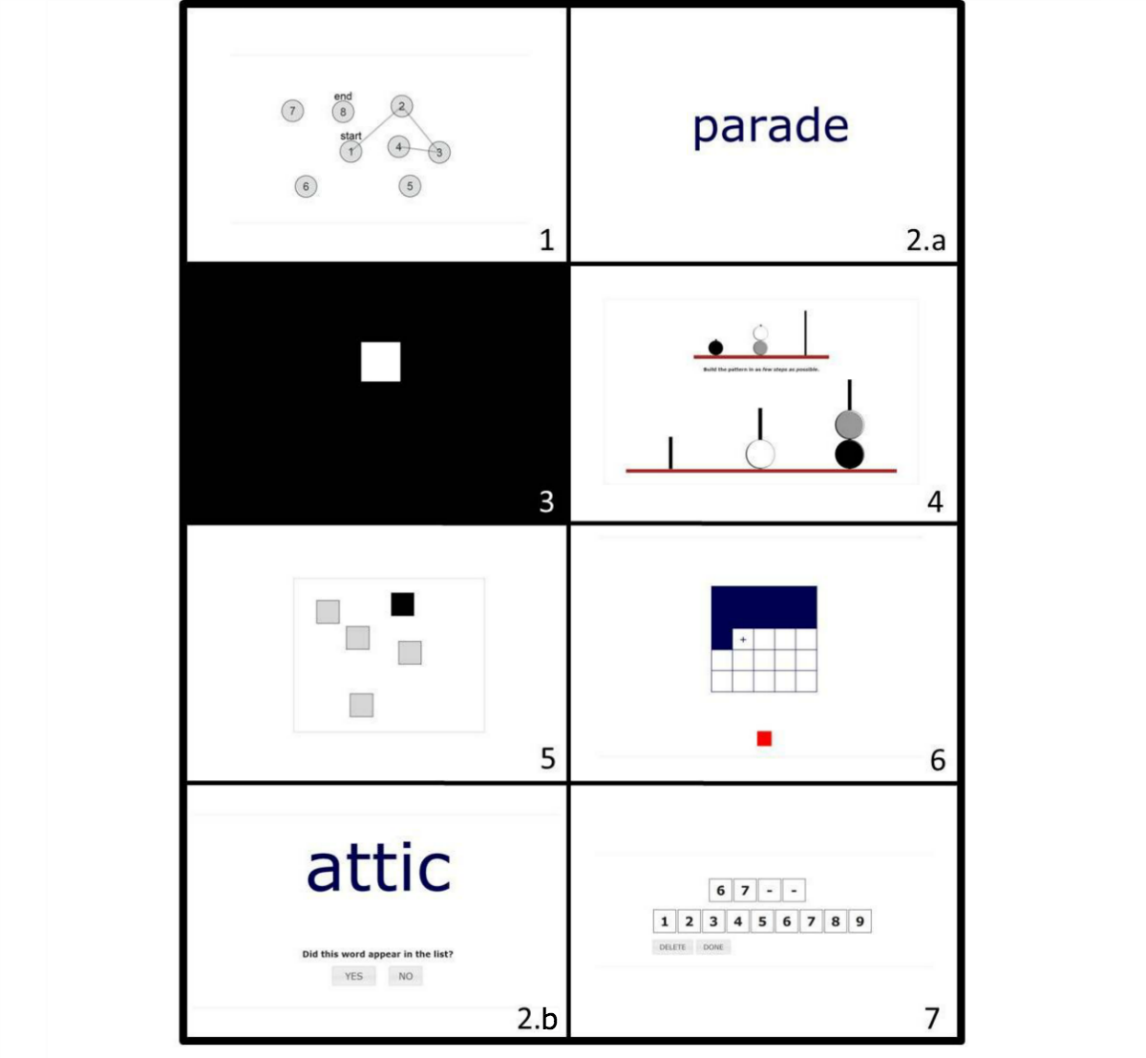
Neuropsychological tests of the Amsterdam Cognition Scan, in chronological order.

The neuropsychological assessment consisted of seven tests that provide 12 measurements on generic cognitive ability areas, including a composite score. All tests were based on conventional traditional neuropsychological tests and developed to measure similar cognitive measurement constructs. An overview of these neuropsychological tests, their measurement constructs, and the traditional test versions are shown in Table 1; a more detailed description of these tests is provided in Multimedia Appendix 1; and Figure 2 and Multimedia Appendix 2 provide screenshots of the different elements of the ACS. In chronological order, the following tests were assessed: Connect the Dots I and II, Wordlist Learning, Reaction Speed, Place the Beads (analyses on this test are based on the Place the Beads reference sample), Box Tapping, Fill the Grid, Wordlist Delayed Recall and Recognition, and Digit Sequences I and II. Tests were developed for online self-administration. For participants, all that was required was a computer with sound and an Internet connection. One could use a mouse or touchpad to respond, as preferred. The ACS was programmed to run on all major Internet browsers and on all common variations in operating systems, which means that no downloads were required to access or complete the battery. Every neuropsychological test started with an instruction video in which screen captures of the test, combined with voiceover instructions, were presented. Subsequent practice with feedback ensured that participants understood the instructions and were ready to take the actual test. The ACS was presented in Dutch but is available in English as well. A German, French, and Spanish version is currently being developed.

**Table 1:** Tests of the Amsterdam Cognition Scan and their equivalent traditional tests.

Online tests^a^	Test domains	Main outcome measure	Traditional equivalent
1. Connect the Dots I; Connect the Dots II	Visuomotor tracking, planning, cognitive flexibility, divided attention	Completion time (I and II)	Trail Making Test A Trail Making Test B [30]
2.a. Wordlist Learning	Verbal learning	Total number of correct words (trial 1 to 5)	15 Words test (Dutch version of Rey Auditory Verbal Learning test) [31]
3. Reaction Speed	Information processing speed and attention	Mean reaction time	Visual Reaction Time (subtest FePsy) [32]
4. Place the Beads	Planning, response inhibition, visuospatial memory	Total number of extra moves	Tower of London, Drexel University (TOL-dx) [33]
5. Box Tapping	Visuospatial short-term memory	Total number of correctly repeated sequences	Corsi Block-tapping Test [34]
6. Fill the Grid	Fine motor skills	Completion time	Grooved Pegboard [35]
2.b. Wordlist Delayed Recall & Recognition	Retention of information: free recall and recognition	Total number of correct words; free recall and recognition	15 Words test
7. Digit Sequences I; Digit Sequences II	I: attention II: working memory	Total number of correctly repeated sequences (I and II)	WAIS III Digit Span (forward and backward) [36]

^a^Online tests displayed in chronological order

### Data Analysis

Outliers on the neuropsychological test scores were identified and excluded from data analyses to limit the influence of extreme scores, possibly reflecting periods of participant distraction or technical glitches. For reaction time outcomes (Connect the Dots, Reaction Speed, and Fill the Grid), we used the median absolute deviation (MAD) method [37] to detect outliers. MADs were calculated and applied times 3.5 separately per age group: ≤40 years, 41-59 years, and ≥60 years to indicate upper and lower data limits. For tests that rely on the number of correct responses and for which zero scores are more likely to reflect usability issues than true (floor) performance (Wordlist Learning, Box Tapping, and Digit Sequences), zero scores were considered outliers and excluded from analyses.

A composite score for the total online neuropsychological test battery (total score) was calculated by averaging the standardized scores of the main outcome measures Connect the Dots I and II, Wordlist Learning, Reaction Speed, Box Tapping, Fill the Grid, Wordlist Delayed Recall, and Digit Sequences I and II. Standardized scores of Connect the Dots, Reaction Speed, and Fill the Grid were reversed scored (z-score times −1) first. The Place the Beads test was not included in the composite score because this test was completed by a different group of participants. Wordlist Recognition was not included in the composite score as there was very little variance in the data.

To assess practice effects, paired sample *t* tests on the differences between performance on the first and the second assessments were performed. A significant (*P*<.05) improvement over time was interpreted as indicating practice effects [38].

Test-retest reliability was assessed using the intraclass correlation coefficient (ICC): two-way mixed effects model with measures of absolute agreement (95% CI). Similar to other correlation measures, higher ICC values indicate less error variance and better test-retest reliability, but unlike other measures, ICCs take both random and systemic error (eg, practice effects) into account when calculating reliability [39,40]. Generally, as a criterion for acceptable test-retest reliability, ICC values of .60 or .70 and higher are recommended [39,41-43]. In agreement with these recommendations, we used a criterion of .60 to indicate which tests have acceptable reliability results and which do not. To enable interpretation of reliability results and comparisons with reported test-retest reliabilities in the literature, Pearson *r* and Spearman rho (depending on the distribution of scores on the particular measurements) were also calculated.

An absolute measure (in the unit of the measurement instrument) of reliability was calculated by the SE of measurement (SEM), using the mean squared error (MS; residual) term from the ICC formula as shown in equation (1):

(1) SEM = √MS
_residual_

In addition, the smallest detectible change (SDC), indicating—similar to the reliable change index—an interval for change beyond measurement error, was calculated as shown in equation (2) [39]:

(2) SDC = 1.96 × √2 × SEM

When applying the SDC to group scores (eg, for research purposes), averaged scores make the measurement error smaller. Therefore, to indicate real change of group mean scores, SDC was divided by the square-root of the sample size, as shown in equation (3) [44]:

(3) SDC
_group_ = √n
_sample_

Multiple regression analyses (MRA) were performed on Connect the Dots I and II, Wordlist Learning and Delayed Recall, Reaction Speed, Place the Beads, Box Tapping, Fill the Grid, Digit Sequences I and II, and the ACS total score to explore sensitivity to demographic variables. To do so, first, reference group raw scores were converted into normalized and standardized scores (mean 0, SD 1). Reverse scoring was applied for Connect the Dots, Reaction Speed, and Fill the Grid. For Connect the Dots I, Reaction Speed, and Fill the Grid, inverse transformations were applied (1 / [reversed] test score), whereas for Connect the Dots II a log10 transformation and for Place the Beads, a squared root transformation was applied. Next, for all measures, we regressed standardized scores on the predictive variables age, age-squared, gender (0=female, 1=male), and education (Verhage education scores [45]) entered blockwise. These variables were selected based on literature [25,26,46] and on previous findings of influencing factors on ACS performance in a sample of cancer patients [22]. Age was centered (age – mean group age) to avoid multicollinearity with the quadratic age term [47], which was added to model nonlinear relationships between age and test performance [26,48]. The Verhage education score, which ranges from 1 to 7, was transformed to a high-low score: 0=high (Verhage 6 and 7) and 1=low or medium (Verhage 1, 2, 3, 4, and 5). Low and medium levels were merged to one level because the lowest level was represented by only one participant with Verhage 3. In addition, for explorative analyses on the influence of computer skills and experience, we ran all models with tested computer skills as supplementary predictor and performed correlational analyses on self-reported mean number of hours of computer use per week (computer use indicated as [1] 0-5, [2] 5-15, [3] 15-35, or [4] >35 hours a week). Model parameters—consisting of standardized and unstandardized beta weights for each predictor and a predictive constant—and the SD of residuals of the participant’s observed scores were estimated for each outcome measure.

Next, nonsignificant predictors (*P*>.05) were excluded from the models, and MRA were rerun. The resulting model parameters provided the basis for regression-based formulas that can be used to calculate demographically adjusted scores.

To check assumptions for MRA, multicollinearity was assessed by calculating the variance inflation factors (VIFs) and checking whether any values were >10 [49]. In addition, for potential future interpretation of CIs and statistical significance, homoscedasticity was evaluated by visual inspection of residual-predicted values scatter plots, and normal distribution of the residuals was evaluated by visual inspection of residual histograms and p-p plots.

All statistical analyses were performed using SPSS Statistics for Windows, version 22.0 (IBM Corp). For reliability analyses, probabilities of *P*<.01 (two-tailed) were considered statistically significant to reduce chance of type one error. For multiple regressions and analyses on practice effects, probabilities of *P*<.05 (two-tailed) were considered statistically significant to be more conservative.

## Results

### Main Findings

**Figure 3:**
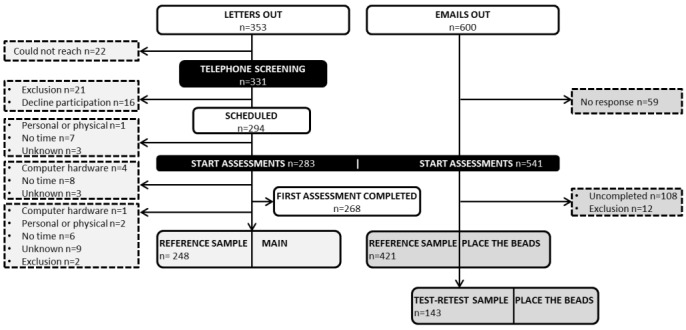
Participation and completion rates of the main reference sample and the Place the Beads reference sample.

For the main reference sample, letters were sent to 353 people (friends and family of patients from the ACS validation study), of whom 294 (83.3%) agreed to participate. Of these 294 participants, a total of 250 (70.8% of all 353 invitees) completed both assessments. A total of 11 participants dropped out before the start of the study and 33 during the study. The data of 2 participants were excluded from analyses because of missing demographic information (age and level of education). Therefore, analyses are based on 248 participants. In addition, 6 participants were excluded from analyses on the Wordlist Learning test, as they indicated to have used a notepad during one or both assessments. One participant was excluded from analyses on Digit Sequences II, as this participant used the entry field to memorize the digits before entering them in reverse order. Table 2 shows demographic and medical characteristics (history of brain pathology and medication use) of the dropout group and of the reference sample (n=248). All participants of the main reference sample were in the age range of 19 to 81 years (mean 49.1, SD 12.9), and 63.3% (157/248) were female. Seventy-one percent of the participants had high education levels (Verhage scale 6 or 7). For the Place the Beads reference sample, about 600 people (subscribers to an online respondent panel) were invited by email, of which 541 (90.2%) opened the link to the online test. Of these 541 participants, 433 (72.2% from all 600 invitees) completed the first assessment. A total of 12 participants were excluded from analyses as there was no information available on their gender. The 421 participants of the final Place the Beads sample were in the age range of 18 to 78 years (mean 47.9, SD 13.8), and 251 59.6% (251/421) were female. A subgroup of 143 Place the Beads participants also completed a second assessment for test-retest analyses. See Table 2 for an overview of demographic characteristics of these subgroups of the Place the Beads samples. Figure 3 illustrates participation and completion rates of the main reference sample and the Place the Beads reference sample.

### Conditions During the Self-Administered Assessments

The vast majority (75.4%, 187/248) of the participants used a standard mouse, 19% (47/248) used a trackpad, and 5.6% (14/248) used something else (eg, joystick or pen mouse) at the first assessment. Furthermore, most participants used the same device type at the second assessment (92.7%, 230/248). The results of the online debriefing on the first assessment with the ACS (n=235) are displayed in Table 3. Main findings are that 13.6% (32/235) participants were disrupted, and 11.5% (27/235) participants experienced some type of technical problem during the assessment; mostly problems with the Internet connection (23/235, 9.8%) and a few cases of problems with hardware (sound system or failed attempts having tried to use a tablet; 1.7%, 4/235). Technical problems were in all cases resolvable and did not prevent participants from completing the assessment. By far, most participants preferred online assessments from home over online or traditional assessments from the hospital (97.3% out of n=225 answering this question).

### Test-Retest Reliability and Practice Effects

Test-retest reliability results indicated statistically significant correlations between the first and the second assessment for all 12 outcome measures (all *P*<.001; see Table 4). HADS and MFI-20 questionnaire results are presented separately (see Multimedia Appendix 3). The ICCs of the individual tests ranged from .45 (Place the Beads) to .80 (Fill the Grid). The total score had an ICC of .83. The majority of measures had ICCs above our .60 criterion (Connect the Dots I and II, Wordlist Recognition, Reaction Speed, Fill the Grid, Digit Sequences II, and the total score), except for Wordlist Learning and Delayed Recall, Place the Beads, Box Tapping, and Digit Sequences I.

**Table 2:** Demographics and clinical characteristics of the main reference sample and the Place the Beads reference sample.

Characteristics			Dropouts^a^ (n=44)	Main reference sample (n=248)	Place the Beads reference sample (n=421)	Place the Beads test-retest subsample (n=143)
**Demographics**						
	**Gender, n (%)**					
		Female	25 (57)	157 (63.3)	251 (59.6)	65 (45.5)
		Male	19 (43)	91 (36.7)	170 (40.4)	78 (54.5)
	Age (years), mean (SD)		44.4 (14.1)	49.2 (13)	47.9 (13.8)	48.3 (12.7)
	**Education level^b^, n (%)**					
		Low	—	—	8 (1.9)	2 (1.4)
		Medium	11 (25)	73 (29.4)	196 (46.6)	64 (44.8)
		High	33 (75)	175 (70.6)	217 (51.5)	77 (53.8)
	Computer experience (years), mean (SD)		19.9 (7.2)	19.5 (6.3)	—	—
**Clinical characteristics, n (%)**						
	Dyslexic		3 (7)	4 (1.6)	—	—
	Concussion (history)		11 (25)	59 (23.8)	—	—
	Whiplash (history)		1 (2)	10 (4)	—	—
	Stroke (history)		0	1 (0.4)	—	—
	Transient ischemic attacks (history)		0	1 (0.4)	—	—
	Heart disease (current treatment)		1 (2)	5 (2)	—	—
	High blood pressure (current treatment)		0	28 (11.3)	—	—
	Diabetes mellitus (current treatment)		1 (2)	6 (2.4)	—	—
	Sleep medication		0	5 (2)	—	—
	Antidepressants		2 (5)	8 (3.2)	—	—
	Painkillers		0	5 (2)	—	—

^a^Dropouts from the group of 296 people who agreed to participate as part of the main reference sample: 11 dropped out before starting the assessments, 15 dropped out after starting—but not completing—the first assessment, and 18 dropped out after completing the first assessment. Of other nonparticipants (n=59), no characteristics were available because of the recruitment procedure.

^b^Education is based on Verhage education scores 1 to 7 [45], corresponding with the following US years of education: 1: 1 to 5 years, 2: 6 years, 3: 7 to 8 years, 4: 7 to 9 years, 5: 7 to 10 years, 6: 7 to 16 years, and 7: 17 to 20 years. Low=Verhage 1 or 2; medium=Verhage 3, 4, or 5; and high=Verhage 6 or 7.

**Table 3:** Online debriefing on conditions during first assessments with the Amsterdam Cognition Scan (n=235).

Question		Frequency (%)
Others present during assessment		40 (17)
Task unclear after practice session		0 (0)
Received help from others		1 (0.4)
Used aid^a^		4 (1.7)
**Were disrupted**		32 (13.6)
	By telephone or doorbell	14 (6)
	By house members	17 (7.2)
**Experienced technical problems**		27 (11.5)
	Internet connection	23 (9.8)
	Hardware problem	4 (1.7)
**Computer use a week (hours)**		
	0-5	49 (20.9)
	5-15	62 (26.4)
	15-35	84 (35.7)
	>35	40 (17)

^a^In all cases, this concerned paper and pencil use.

In addition, we found Pearson and Spearman (depending on score distributions) correlations highly comparable with the highest test-retest correlations of the traditional tests reported in literature on studies with similar retest intervals (2 weeks-6 months). For Wordlist Delayed Recall, we found the reliability coefficient to be somewhat lower than in the literature but still relatively high (ρ=.64 vs *r*=.80) [50].

On the basis of the SEM, the SDC was calculated for use with mean group results for all neuropsychological outcome measures (see Table 4). Paired samples *t* tests showed significant practice effects for the following tests: Connect the Dots I and II (both *P*<.001), Wordlist Learning and Delayed Recall (both *P*<.001), Place the Beads (*P*=.002), Box tapping (*P*<.001), and Fill the Grid (*P*<.001). A ceiling effect was observed for the Wordlist Recognition measure, indicated by small SDs and no improvement over time. For the seven measures that showed significant performance change over time, the difference scores (mean 1 – mean 2) were added to the group SDC to account for practice effects.

**Table 4:** Test-retest reliability and practice effects (significant *t* values in italics) for the Amsterdam Cognition Scale (Total participants [N]=248). ICC: intraclass correlation coefficient; N/A: not applicable; SDC: smallest detectable change; SEM: standard error of measurement.

Test	n (%)	Mean 1 (SD)	Mean 2 (SD)	*t* value (degrees of freedom), *P* value	SEM	SDC group^a,b^	ICC^c^	Correlation coefficient^c^	Literature^d^
Connect the Dots I	240 (96.8)	34.83 (9.39)	32.48 (7.79)	*5.43* (239), <.001	4.76	*1.00*	.67	.75^e^	.55-.73
Connect the Dots II	246 (99.2)	58.05 (18.45)	54.95 (17.62)	*3.6* (245), <.001	9.55	*1.89*	.71	.74^e^	.56-.79
Wordlist Learning	241 (97.2)	52.02 (10.13)	58.11 (9.80)	−*12.31* (240), <.001	5.44	*1.36*	.59	.75^e^	.80
Wordlist Delayed recall	241 (97.2)	11.15 (2.71)	12.09 (2.88)	−*5.34* (240), <.001	3.69	*0.72*	.50	.64^e^	.83
Wordlist Recognition	242 (97.6)	44.17 (1.57)	44.31 (1.72)	−1.76 (241), .08	0.90	0.16	.70	.54^e^	.48
Reaction Speed	241 (97.2)	308.88 (44.57)	310.49 (44.85)	−0.69 (240), .49	25.60	4.60	.67	.74^e^	.20-.82
Place the Beads^f^	143 (100)	25.63 (14.03)	21.54 (15.81)	*3.17* (142), .002	11.15	*2.93*	.45	.50^e^	.38-.70
Box Tapping	232 (93.5)	9.17 (2.26)	9.70 (1.91)	−*3.91* (231), <.001	1.47	*0.30*	.49	.46^e^	.42-.79
Fill the Grid	241 (97.2)	62.59 (12.69)	60.73 (11.42)	*3.85* (240), <.001	5.30	*0.95*	.80	.81^e^	.72-.86
Digit Sequences I	245 (98.8)	10.40 (2.22)	10.61 (2.17)	−1.52 (244), .13	1.49	0.28	.54	.54^g^	.61-.78
Digit Sequences II	242 (97.6)	8.50 (2.72)	8.78 (2.87)	−1.81 (241), .07	1.68	0.32	.64	.64^g^	.46-.71
Total score^h^	206 (83.1)	0.06 (.53)	0.07(.55)	−0.23 (205), .82	0.05	0.01	.83	.83^g^	N/A	

^a^SDC group: SDC / √n.

^b^For measures with a significant practice effect (*t* value in italics), Mean1 – Mean2 difference scores were added.

^c^All correlation coefficients were significant at *P*<.001.

^d^Range of Pearson r correlation coefficients as found in the literature on studies with retest intervals between 2 weeks and 6 months on adults without progressive disease is presented: Trail Making Test A [51-54]; Trail Making Test B [23,53,54]; 15 Word Test Learning and Delayed Recall [50]; 15 Word Test Recognition [55]; Visual Reaction Time FePsy [55,56]; Tower of London [23,56-58]; Corsi Block-tapping [52,57,59,60]; Grooved Pegboard [61,62]; WAIS Digit Span [36,63].

^e^Spearman ρ.

^f^Analyses performed on data from the Place the Beads sample; participants that completed both assessments only (n=143).

^g^Pearson *r*.

^h^Total score is based on mean standardized scores from Connect the Dots I and II, Wordlist Learning and Delayed Recall, Reaction Speed, Box Tapping, Fill the Grid, and Digit Sequences I and II.

### Influence of Age, Gender, Education, and Computer Skills on Neuropsychological Test Scores

The multiple regression models—all including the factors age, age-squared, gender, and/or education if significant (*P*<.05)—are presented in Table 5. MRA, first, showed that higher age significantly deteriorated scores on all neuropsychological tests, as well as on the total score (see betas in Table 5). In addition to this linear age effect, a negative quadratic age effect was found for Connect the Dots II and the total score, indicating that age deteriorated scores on these measures increasingly with older age. Second, men outperformed women on Connect the Dots I, Reaction Speed, Box Tapping, Fill the grid, and the total score. Women outperformed men on Wordlist Delayed Recall. Finally, participants with low or medium education showed lower test scores than participants with high education on Place the Beads, Digit Sequences I and II, and the total score. Mean scores and SDs for all outcome measures stratified by gender and age are provided in Table 6. MRA performed only on participants who used a standard mouse (thus excluding participants who used a trackpad or “other” device) yielded highly similar results; the effect of demographic characteristics were similar, except for age no longer being significant for performance on Digits I and gender no longer being significant for performance on Wordlist Learning and Reaction Speed.

**Table 5:** Multiple linear regression models including the factors age, age-squared, gender, and/or education if significant (*P*<.05) for Amsterdam Cognition Scan outcome measures. All multiple regression analyses (MRA) are performed with normalized and standardized (mean 0, SD 1) scores. Education: 0=high, 1=low or medium; Gender: 0=female, 1=male.

Test	Variable	Beta	SE beta	Standard beta	*t* value	*P* value	R^2^	SD (residual)
Connect the Dots I^a,b^	Constant	−.149	.066		2.26			
	Age	−.042	.004	.549	10.44	<.001		
	Gender	.401	.109	-.193	-3.68	<.001	0.324	0.819
Connect the Dots II^a,c^	Constant	.101	.067		1.51				
	Age	−.045	.004	−.580	-11.06	<.001		
	Age-squared	−.001	.000	−.126	-2.40	.02	0.33	0.815
Wordlist Learning	Constant	−.001	.060		-.02			
	Age	−.027	.005	−.346	-5.78	<.001	0.116	0.938
Wordlist Delayed Recall	Constant	.102	.077		1.32				
	Age	−.018	.005	−.234	-3.79	<.001		
	Gender	−.282	.128	−.136	-2.21	.03	0.068	0.961
Reaction Speed^a,b^	Constant	−.106	.078		1.35			
	Age	−.019	.005	.248	3.98	<.001		
	Gender	.272	.128	−.132	-2.12	.04	0.067	0.962
Place the Beads^a,d,e^	Constant	.109	.067		1.63				
	Age	−.014	.004	−.195	-3.96	<.001		
	Education^f^	−.226	.098	−.113	-2.30	.02	0.058	0.968
Box Tapping	Constant	−.145	.077		-1.90			
	Age	−.023	.005	−.298	-4.86	<.001		
	Gender	.372	.128	.179	2.91	.004	0.11	0.939
Fill the Grid^a,b^	Constant	−.144	.069		-2.08			
	Age	−.040	.004	−.505	-9.18	<.001		
	Gender	.358	.114	.173	3.15	.002	0.267	0.852
Digit Sequences I	Constant	.103	.074		1.39			
	Age	−.010	.005	−.130	-2.08	.04		
	Education	−.350	.137	−.160	-2.56	.01	0.036	0.978
Digit Sequences II	Constant	.100	.074		1.35			
	Age	−.015	.005	−.196	-3.15	<.001		
	Education	−.357	.137	−.163	-2.61	.02	0.06	0.966
Total score	Constant	.068	.044		1.56			
	Age	−.025	.002	−.597	-11.04	<.001		
	Age-squared	.000	.000	−.117	-2.16	.03			
	Gender	.120	.057	.111	2.10	.02		
	Education	−.129	.061	−.112	-2.11	.03	0.351	0.420

^a^Reverse scoring was applied before MRA.

^b^Inverse transformations were applied (eg, 1/Connect the Dots I).

^c^Log10 transformation was applied.

^d^Squared root transformation was applied.

^e^Analyses performed on data from the Place the Beads sample.

^f^Note that education levels were more balanced in the Place the Beads sample by including more participants with middle or lower education than in the main sample.

**Table 6:** Mean raw scores and SDs for the Amsterdam Cognition Scan measures stratified by gender and age.

Test	Age (years)	Gender	
		Female, mean (SD)	Male, mean (SD)
Connect the Dots I	≤40	31.28 (6.8)	26.15 (4.5)
	41-59	34.86 (8.6)	33.16 (6.9)
	≥60	46.55 (13.8)	39.84 (10.8)
Connect the Dots II	≤40	47.56 (12.9)	48.2 (12.7)
	41-59	55.6 (14.9)	54.41 (11.55)
	≥60	79.17 (22.8)	68.4 (18.07)
Wordlist Learning	≤40	56.76 (8.1)	55.45 (9.2)	
	41-59	53.96 (8.8)	50.09 (9.8)
	≥60	48.03 (13)	46.26 (9.3)
Wordlist Delayed Recall	≤40	12.14 (1.8)	11.95 (2.6)
	41-59	11.54 (2.5)	10.44 (3.2)
	≥ 60	10.53 (2.9)	9.57 (3.4)
Wordlist Recognition	≤40	44.65 (0.7)	44.59 (0.96)
	41-59	44.31 (1.5)	43.93 (1.9)
	≥60	43.67 (2)	43.78 (1.5)
Reaction Speed	≤40	304.4 (35)	289.7 (33)
	41-59	309.2 (47)	297.9 (38)
	≥60	336.1 (57)	321.5 (41)
Place the Beads^a^	≤40	23.49 (14.1)	21.68 (15.5)
	41-59	27.3 (13.3)	27.07 (15.4)
	≥60	35.47 (17.7)	28.24 (15.3)
Box Tapping	≤40	9.38 (1.8)	10.24 (1.7)
	41-59	8.94 (2.6)	9.67 (2)
	≥60	7.85 (2.1)	8.78 (2.7)
Fill the Grid	≤40	60 (10.3)	50.21 (8.3)
	41-59	62 (11.7)	60.7 (10.7)
	≥60	73.1 (12.2)	72.36 (14.8)
Digit Sequences I	≤40	10.73 (2.1)	10.45 (2.6)
	41-59	10.14 (2.5)	11.02 (2)
	≥60	10.14 (1.9)	9.96 (2.3)
Digit Sequences II	≤40	9.22 (2.9)	9.32 (2.9)
	41-59	8.45 (2.7)	8.98 (2.4)
	≥60	7.23 (2.7)	8.17 (2.6)
Total score^b^	≤40	2.8 (3.7)	3.88 (3.9)
	41-59	0.64 (4.2)	1.18 (3.38)
	≥60	-4.22 (4.8)	-3.5 (4.2)

^a^Analyses performed on data from the Place the Beads sample.

^b^Total score is based on standardized score of all main neuropsychological outcome measures, except for Wordlist Recognition and Place the Beads.

To further investigate the widespread association between cognitive performance and gender, explorative analyses were performed. These analyses showed no general age difference between women and men (*t*_246_=−0.79, *P*=.43). Level of education was distributed somewhat differently, with more very highly educated (Verhage 7) men than women (χ²_4_[n=248]=9.62, *P*=.047). Moreover, on average, men had more years of self-reported computer experience (*t*_246_=−3.07, *P*=.002) and higher scores on the computer skills compound score (*t*_232,1_=−2.07, *P=*.04). Concerning tested computer skills, when looking into gender differences between young (≤40 years), middle aged (41-59 years), and older aged (≥60 years) participants separately, only in the younger age group, men significantly outperformed women (*t*_57_=−2.07, *P*=.04). We entered both self-reported computer experience and tested computer skill scores as additional predictors in the MRA but removed self-reported computer experience as it was not associated with any of the neuropsychological outcome measures. Tested computer skills, on the other hand, proved to be associated with performance on all time-based measures (Connect the Dots I and II, Reaction Speed, and Fill the Grid) and the total score (see Multimedia Appendix 4). For most of these measures (Connect the Dots I, Reaction Speed, Fill the Grid, and the total score), a beneficial effect of being male was found in the main MRA. After entering computer skills as a predictor, gender was no longer significantly associated with Connect the Dots II, Reaction speed, and the total score, but it remained significantly associated—albeit less so—with Connect the Dots I and Fill the Grid. Additionally, a second self-reported measure of computer experience—“mean number of hours computer use per week”—was positively correlated with several ACS measures: Connect the Dots I (ρ=.37; *P*<.001), Connect the Dots II (ρ=.27; *P*<.001), Wordlist Learning (ρ=.27; *P*<.001), Reaction Speed (ρ=.16; *P*=.02), Box Tapping (ρ=.33; *P*<.001), Fill the Grid (ρ=.36; *P*<.001), and the total score (ρ=.33; *P*<.001), as well as with tested computer skills (ρ=.4; *P*<.001).

There was no proof of multicollinearity (the highest VIF value was 1.06), indicating statistical independence of the proposed predictor variables. Evaluation of the p-p normality plots of the final models showed that standardized residuals were not normally distributed for Wordlist Delayed Recall and Box Tapping, and evaluation of residual-predicted values scatter plots indicated heteroscedasticity for Connect the Dots II. For the current analyses—focusing on estimation of model parameters—lack of normality and homoscedasticity does not invalidate results [64].

### Regression-Based Normative Data

The regression models, as presented in Table 5, provide regression-based normative data. First, for each measure, demographically based predicted scores can be calculated using the constant value and the unstandardized beta weights. Age is centered by calculating the difference between the age of the patient and the mean age of the reference sample (49.19 years). For example, on Connect the Dots I, a woman aged 55 years would have a predicted score of −.149 + (55-49.19)(−.042) + 0(.401)=−.39. As we used normalized and standardized scores for the initial multiple regression analyses, actual scores need to be normalized and standardized using the same transformations as well. Subsequently, by calculating the standardized difference between actual and predicted score, a demographically corrected norm score is established. If the woman of our example has an actual score of 60 seconds, this corresponds to a normalized (in the case of Connect the Dots I reverse transformations were applied) and standardized score of ([1/60]-.0304)/.00793=−1.73, resulting in a difference score of −.39-(−1.73)=−1.34 and a norm score of −1.34/.819=−1.64.

## Discussion

On the basis of assessments from 248 healthy Dutch adults, we studied psychometric properties and established regression-based, demographically corrected, normative data for the ACS.

### Test-Retest Reliability and Practice Effects

Test-retest reliability was found to be adequate for most tests. ICCs (>.60) indicated sufficient consistency over time for 7 out of 12 outcome measures. Consistency between overall ACS performance (total score) was especially high. This could be expected as averaged scores generally generate higher correlations than single measurements [38,39]. Test-retest correlations were highly comparable with the highest test-retest correlations for equivalent traditional tests as reported in the literature. Current reliability results were also comparable with the test-retest results on the ACS as observed in a sample of cancer patients (6 week interval; n=96) [22], although we currently find higher reliability for Reaction Speed, Box tapping, and Fill the Grid. An important difference between this study with healthy subjects and our previous study with cancer patients was the degree of variability in computer hardware. In the patient study, repeated testing was performed from two different settings (home and hospital, in a counterbalanced design) and thus, on two different computers; in this study, the majority of participants performed both assessments on the same computer. As Reaction Speed, Box tapping, and Fill the Grid are highly dependent on mouse input, it is likely that consistent hardware over assessments has improved test-retest reliability. Therefore, in future applications with repeated testing, it will be important to pursue consistency of computer configuration; preferably using one computer and one browser type.

We found significant differences between the first and the second assessment on most of our tests (all measures except for Digit Sequences I and II, Reaction Speed, and Wordlist Recognition). For this last test, a ceiling effect was found, as is commonly found in the literature for its traditional equivalent [38,65]. Practice effects are commonly observed with repeated cognitive testing, especially for infrequently practiced modes of response and for memory tests [64]. Therefore, in case of repeated testing (eg, before and after treatment), it is important to take such practice effects into account. Our future research will focus on establishing parallel versions of tests that are most susceptible to practice effects to limit overall occurrence of practice effects. This means that after developing parallel versions, psychometric properties will be reevaluated. In this study, we took practice effects into account by adjusting the group SDCs for observed practice effects and by using ICC measures that take systematic error into account for our test-retest correlational analyses.

### Influence of Age, Gender, Education, and Computer Skills on Neuropsychological Test Scores

Considering sensitivity to demographic characteristics, multiple regression-based parameters indicated distinct associations of age, gender, and education with test performance. Age was, as expected, the strongest predictor of cognitive performance [66]. In addition to linear age effects, we found quadratic age effects for two outcome measures (Connect the Dots II and the total score), indicating acceleration of age-related decline with advancing age. Previously, these effects were reported for functions such as verbal memory [25], spatial visualization, processing speed, and reasoning [66,67]. However, in these previous studies and in this study, quadratic age effects are found to be small compared with linear age effects.

A high level of education was predictive for better test performance on only a few outcome measures (Place the Beads, Digit Sequences I and II, and the total score). This suggests that there is limited influence of education level on ACS performance. Note that our main reference sample did not include participants with a low education level, which hampers the assessment of the influence of level of education on cognitive performance. Therefore, MRA should be repeated after collecting reference data from a more heterogeneous reference sample, and information on level of education should preferably be collected in future studies using the ACS.

Gender appeared to be a generic predictor of cognitive performance on the ACS, with men outperforming women on 5 out of 12 measures and women outperforming men on one of the measures. Previous studies have shown more specific effects of gender on cognitive performance; it is commonly found that women outperform men on verbal learning and memory tests, whereas for many other cognitive functions, gender differences are less common [64,68]. To better understand our generic finding of men outperforming women, additional analyses were performed, indicating that computer skills, rather than gender, predicted performance on several gender-sensitive outcome measures. Sensitivity to computer skills may also be influenced by age, as young men in particular were found to outperform women of similar age on computer skills.

We have not yet included our measure of computer skills in the MRA and regression models, as we first want to learn more about its measurement construct and its relation with both online tests and traditional tests. Preliminary data on correlations between tested computer skills and the traditional equivalents of our online neuropsychological tests in a different sample of healthy adults (n=40; 72.5% [29/40] female; mean age 40 years, SD 15.4; 80% [32/40] high education level) indicated associations between our computer skills measure and several traditional neuropsychological tests, mainly in the domains of processing speed and motor coordination. Positive correlations were found for the Trail Making Test (TMT) A (ρ=.5; *P*=.001), 15 Words test (Dutch version of Rey Auditory Verbal Learning test), Learning (ρ=.74; *P*<.001) and Delayed Recall (ρ=.58; *P*=.004), Reaction Speed (ρ=.47; *P*=.002), Grooved Pegboard (ρ=.5; *P*=.001), and the battery total score (ρ=.77; *P*<.001). Moreover, self-reported average number of hours of computer use a week was not associated with performance on traditional neuropsychological tests (except for TMT B: ρ=.34; *P*<.03), whereas in analyses on the online ACS data, this measure was associated with tests on the domains of processing speed and motor coordination (tests that require relatively demanding use of computer input devices). These additional analyses indicate that number of hours computer use is potentially a more valid measure of computer skills than our tests of computer skills, as it did not correlate with offline measures or online measures for which influence of computer skills was not to be expected. Further research on these measures should result in determining an optimal measure of computer skills, which subsequently can be included in the regression-based norms.

### Regression-Based Normative Data

Normative data are established by providing MRA parameters on sensitivity for age, gender, and/or education for all main neuropsychological measures. The MRA parameters constitute formulas that allow for calculating regression-based, demographically corrected norm scores. In addition, to illustrate the influence of the main demographic predictors on cognitive performance, we presented mean test scores stratified by age and gender. Normative data, as presented, are suitable for interpreting ACS results from future studies with Dutch adult populations. American normative data for the English version of the ACS will be available in the near future as well. Concerning future use of the ACS, note that the Wordlist Recognition measure tends to result in a ceiling performance and that the reference data on this test had insufficient variance to enable interpretation of performance. Therefore, currently, Wordlist Learning and Wordlist Delayed Recall are advised to be used as the main measures of verbal learning and memory. Furthermore, as in general response time latencies can differ substantially depending on input device type [69], norm data should ideally be device specific. However, since most people used a standard mouse, we were at present not able to differentiate between devices. In future studies, after collection of additional reference data with a variety of device types, MRA should be repeated.

### Usability

In addition to psychometric properties and normative data, this study showed that the ACS has high usability for cognitive testing with healthy Dutch adults, as was previously found in application with Dutch cancer patients. Virtually all participants completed the test battery from home without additional help. Temporary Internet disconnection occasionally occurred, but this did not prevent participants from completing the ACS successfully. On the basis of the debriefing, we suggest explicitly mentioning in pretest instructions to have a functional sound system, not to use paper-and-pencil, and to strive not to be disrupted during the assessment. This was done in our own instructions but could have been emphasized even more.

### Conclusion

In conclusion, this study with a healthy adult sample shows that the ACS can give reliable measures of various generic cognitive ability areas. For repeated measurements, computer configuration should be consistent. Combined with our normative data that describe which demographic characteristics influence performance, these results allow for initial interpretation of ACS performances. However, to improve the interpretation of the test scores, we will continue to collect reference data, including in a lower educational group and repeat multiple regression analyses after acquiring a more heterogeneous reference sample. We also aim to improve our MRA parameters by performing input device specific calculations and by studying the influence of computer skills and experience and related factors more in depth. The Dutch version of the ACS is estimated to be available for research purposes by the end of 2018. By that time, scoring software based on our normative data will be available as well.

## Appendix

Multimedia Appendix 1 The Amsterdam Cognition Scan—online neuropsychological and computer skill tests.

Multimedia Appendix 2 Screenshots from the Amsterdam Cognition Scan—including in chronological order (1) the general instruction video, (2) computer skill tests, and (3) seven neuropsychological tests with in-between the standardized break animation video.

Multimedia Appendix 3 Questionnaire results on test-retest reliability and practice effects(significant t values in italics).

Multimedia Appendix 4 Multiple linear regression models including the factors age, age-squared, gender, education, and computer skills (tested) for Amsterdam Cognition Scan outcome measures.

## Abbreviations

ACS: Amsterdam Cognition Scan
HADS: Hospital Anxiety and Depression Scale
ICC: intraclass correlation coefficient
MFI-20: Multidimensional Fatigue Inventory-20
MRA: multiple regression analyses
SDC: smallest detectible change
VIF: variance inflation factor
