# Supplementary material for: Online Self-Administered Cognitive Testing Using the Amsterdam Cognition Scan: Establishing Psychometric Properties and Normative Data
Source: J Med Internet Res. 2018 May 30;20(5):e192. doi: 10.2196/jmir.9298 (PMC6000479; doi:10.2196/jmir.9298)
Supplement: Multimedia Appendix 1 [file jmir_v20i5e192_app1.pdf]

Multimedia Appendix 1. The Amsterdam Cognition Scan—online neuropsychological and computer skill tests.

Neuropsychological tests (See Figure 2):

1) Connect the Dots I & II

I: This test measures visuo-motor tracking and planning. Circles, numbered from 1 to 25, are presented on the screen. Participants are instructed to click the numbered circles in increasing order, as quickly as possible and without making mistakes.

II: In combination with part I, this test measures cognitive flexibility and divided attention. Again, 25 circles are presented on the screen. But this time 13 of them are numbered and 12 contained a letter (A to L). Participants are instructed to alternate clicking digits (in increasing numerical order) and letters (in alphabetic order), as fast as possible and without making mistakes.

For both part I and II, whenever the wrong circle is clicked, the circle colors red and participants have to continue clicking in the right sequence. Performance is scored by calculating the total time (from stimulus presentation to completion) for part I and part II, and by calculating the performance time in part I/performance time in part II ratio. Higher scores on part I and II separately indicate slower, and therefore worse, performance.

2.a) Wordlist Learning

This test measures verbal learning. Participants are asked to remember a list of 15 unrelated words. The words have been selected for matching length and frequency with the words from the traditional equivalent test. The words are presented one by one on the screen. After seeing the last word from the list, an entry field appears in which participants can type all the words they remember. The words have to be separated by at least one space, but the order in which the words are entered is not important. Spelling mistakes are programmed to be ignored up to two characters difference with the target word (e.g., the word 'bellooon' would still be considered as referring to the target word 'balloon' and counted as correct). When all the remembered words are entered, participants click on the 'NEXT' button, after which the wordlist is presented again until a fifth presentation. All five

presentations have a different fixed word order. Performance is scored as total number of correctly entered words throughout the five trials. There is a maximum score of 75.

2.b) Wordlist Delayed Recall & Recognition

These tests measure retention of verbal information. The delayed recall of the Wordlist test consists out of a free recall and a recognition task. During the free recall, participants are asked to type all the words they can remember from the previously presented word list once more. There is a maximum score of 15.

During the subsequent recognition task, participants are presented with 45 words on the screen, appearing one at the time. For each word they have to indicate whether or not it was on the list. If a word is recognized as a word from the list, the 'yes'-button has to be clicked. If the word is not recognized, the 'no'-button has to be clicked. Every word requires a response in order to continue. Beside the 15 target words, there are 30 distractor words, which are matched to the target words on semantics, word length, and word frequency. Performance is scored by summing al correct responses (both correctly recognized target words and correctly identified distractor words). There is a maximum score of 45.

3) Reaction Speed

Reaction speed is measured on 30 consecutive trials. Participants start by pressing the mouse button and keeping it pressed down. After a short time (2.5 to 4 seconds), a white block appears on the screen after which, as fast as possible, the mouse button has to be released. The white block always appears on the same place on the screen. Whenever participants let go of the mouse button too early (less than 130 milliseconds after the appearance of the white block), they are instructed to try again. Performance is scored by averaging all correct trials in between 130 and 3950 milliseconds. Higher scores indicate slower, and therefore worse, performance.

4) Place the Beads

This test measures planning and response inhibition. Participants are presented with pictures of two constructions, both consisting of three sticks on which three distinctive beads (a grey, a black, and a white one) are placed. The upper construction is fixed and has to be rebuilt in the lower construction. The aim is to use as few moves as possible to make the lower construction resemble the upper construction. The balls of the lower construction can be moved by dragging them from their current to the desired location. There are two rules presented: 1) only the upper beads can be moved; and 2) no more beads can be placed on a stick than the stick can fit (one, two or three be). Rule-incongruent moves are programmed to be impossible to perform. There are 10 problems to solve. If a problem is not solved within the 2-minute time limit, the test continues with the next problem until all 10 problems have been presented. Performance is scored by summing all moves additional to the minimum number of moves for each problem. If the time limit is reached, the maximum score of 20 is given.

5) Box Tapping

This test measures visuo-spatial short term memory. Participants are presented with a number of blocks on the screen. The blocks blink one by one. Participants are asked to remember the order in which the blocks blinked. When indicated, they must click on the blocks in the same order. After finishing the response, participants click on the 'done'-button; they are then presented with the next series. After two correct series of the same length, the number of blocks is increased by one. This continues until there are 12 blocks on the screen, or whenever there are two incorrect responses within the same series-length. Performance is scored by summing the total number of correctly repeated series.

6) Fill the Grid

This test measures fine motor skills. A grid is presented that consists of 25 squares. Participants are instructed to fill the grid with red blocks as quickly as possible. As soon as the red block appears

underneath the grid, it has to be dragged to the square in which a plus sign is shown. The blocks have to be positioned exactly within the edges of the square. Once a square has been filled correctly, a new red block appears which also has to be placed as fast as possible. The task is completed as soon as all squares are filled. There is a time limit of 3 minutes. Performance is scored by calculating the total time from first to last move. Higher scores indicate slower, and therefore worse, performance.

7) Digit Sequences I & II

I: This test measures attention. A series of digits appear on the screen one by one. Participants have to remember the digits and the order in which they are presented. After the last digit, a digit bar appears on which the digits have to be entered in the correct order. The response is made by clicking on the digits. The selected digits then appear above the bar in the order selected. Mistakes can be corrected by using the 'delete'-button. The response is finished by clicking the 'done'-button, after which the next series appears. After two correct series of the same length, an extra digit is added. The test starts with a series of three digits and halts either after the last series of 12 digits, or whenever there are two incorrect responses within the same series-length. Performance is scored by summing all correctly repeated series.

II: This test measures working memory. The presentation of part II is similar to part I, but this time the digits have to be entered in reverse order. Performance is again scored by summing the total number of correctly repeated series.

Computer skill tests (See Figure 1)

A) Type Skills

This test measures speed and accuracy of keyboard typing. A target sentence is presented which has to be retyped in the response box below. Performance is scored by calculating the total time from first to last key press. Higher scores indicate slower, and therefore worse, performance.

B) Click Skills

This test measures speed of precise clicking via the input device of choice (e.g. mouse or touchpad). A number of circles are presented on the screen, together forming a spiral. From the outside to the center of the spiral, the circles go from large to small. Participants are instructed to click the circles form the outer part of the spiral to the center of the spiral, as quickly as possible and without skipping any of the circles. Performance is scored by calculating the total time from first to last click. There is a time limit of 3 minutes. Higher scores indicate slower, and therefore worse, performance.

C) Drag Skills

This test measures speed of precise drag-and-drop actions. For every trial (eight in total) two elements of the same shape are presented on the screen. One of the elements is black; the other element is white and slightly larger than the black one. Participants are instructed to drag the black element exactly into the white element. After succeeding in doing so, the next trial is presented. Performance is scored by calculating the total time from first to last action. There is a time limit of 3 minutes. Higher scores indicate slower, and therefore worse, performance.
