# Supplementary material for: Online Self-Administered Cognitive Testing Using the Amsterdam Cognition Scan: Establishing Psychometric Properties and Normative Data
Source: J Med Internet Res. 2018 May 30;20(5):e192. doi: 10.2196/jmir.9298 (PMC6000479; doi:10.2196/jmir.9298)
Supplement: Multimedia Appendix 2 [file jmir_v20i5e192_app2.pptx]

## Slide 1
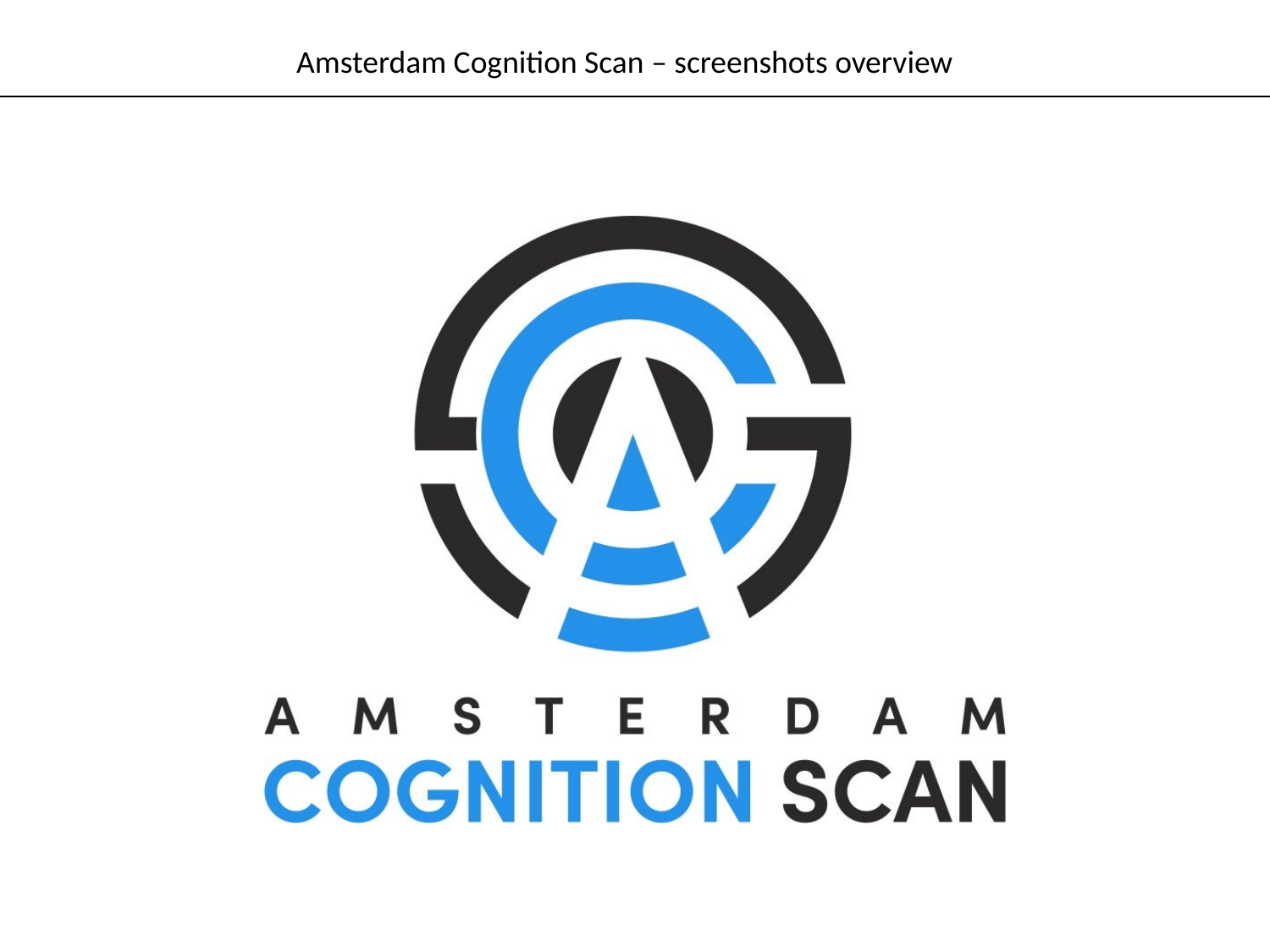

Amsterdam Cognition Scan – screenshots overview

## Slide 2
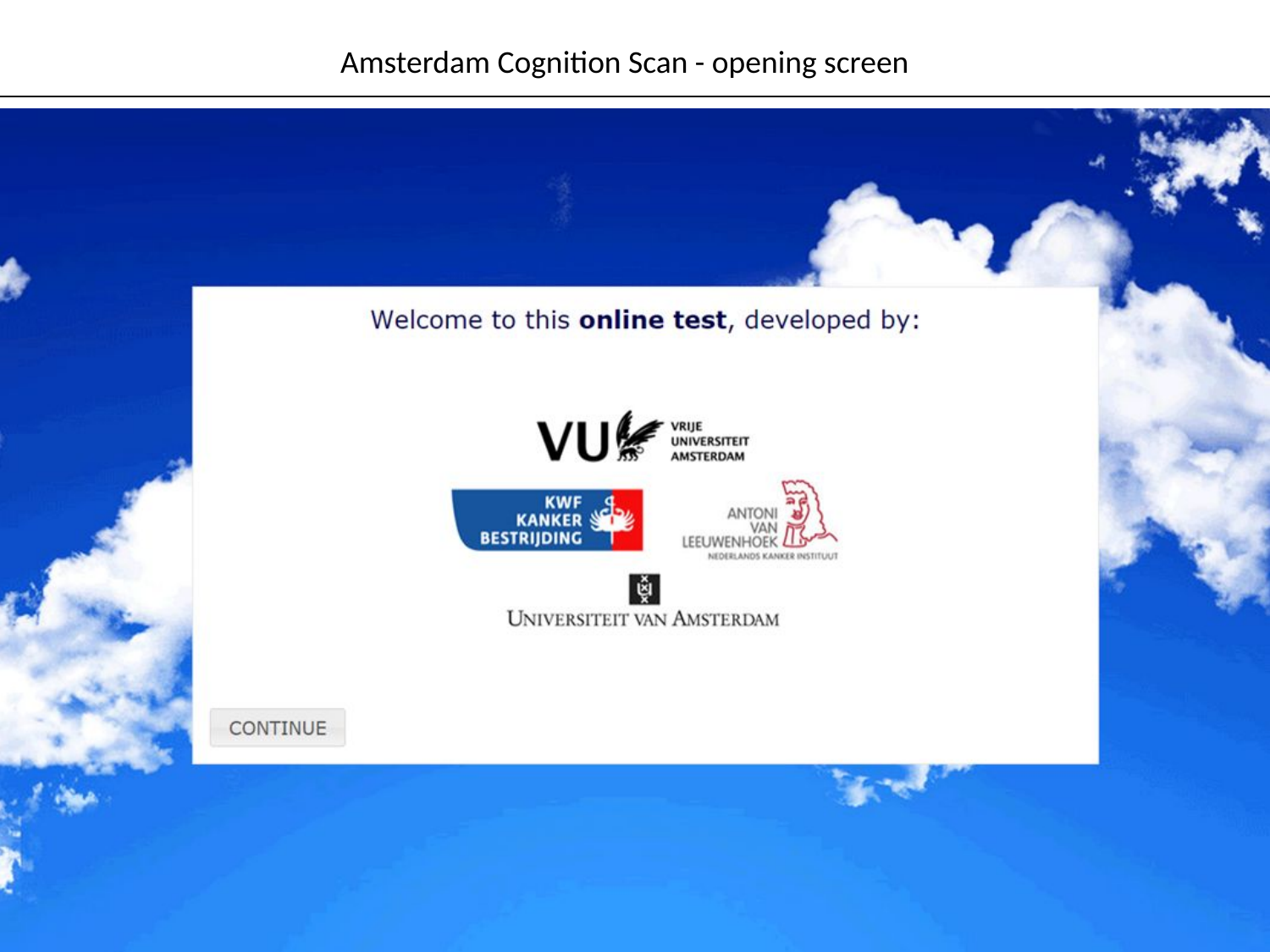

Amsterdam Cognition Scan - opening screen

## Slide 3
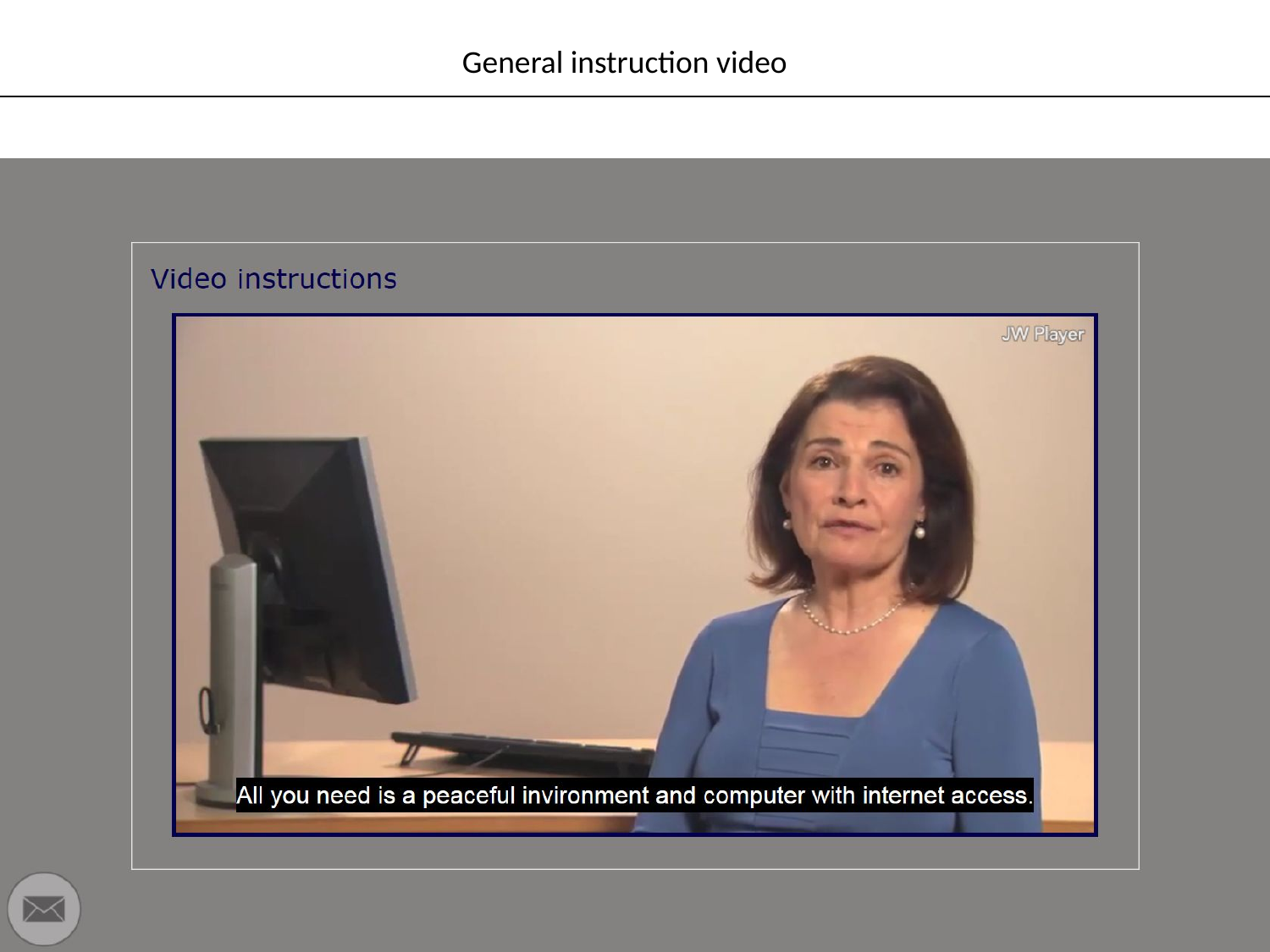

General instruction video

## Slide 4
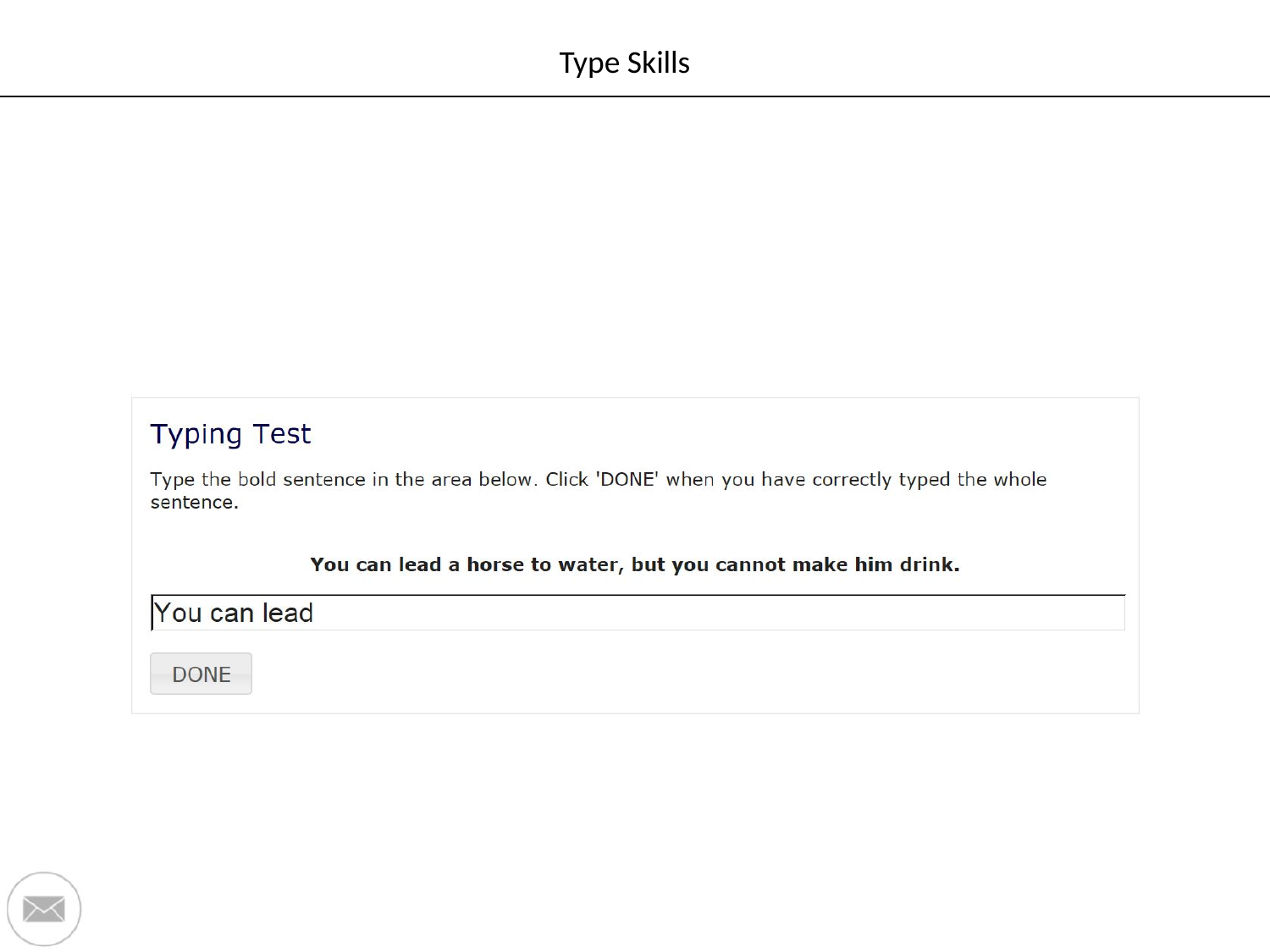

Type Skills

## Slide 5
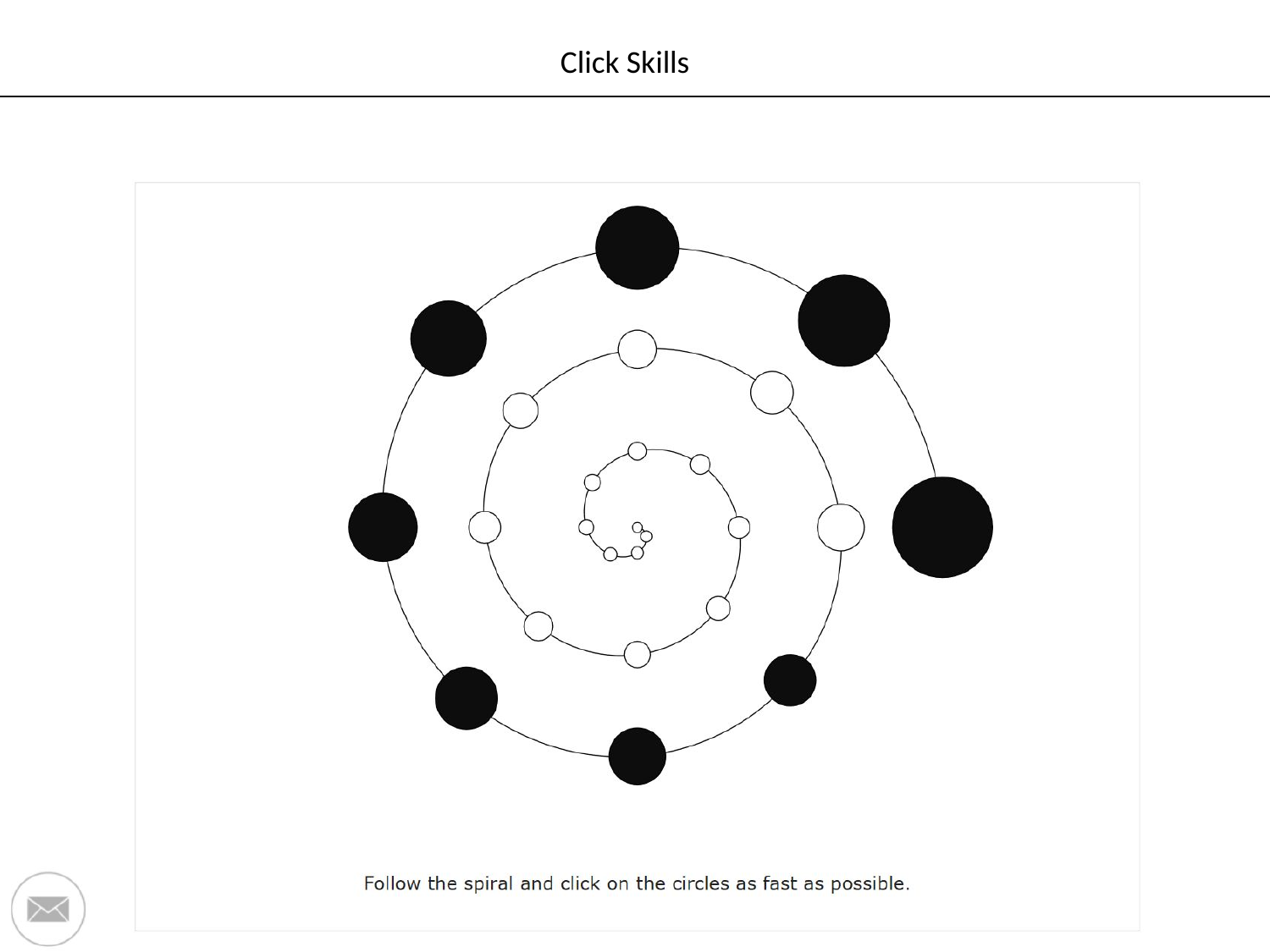

Click Skills

## Slide 6
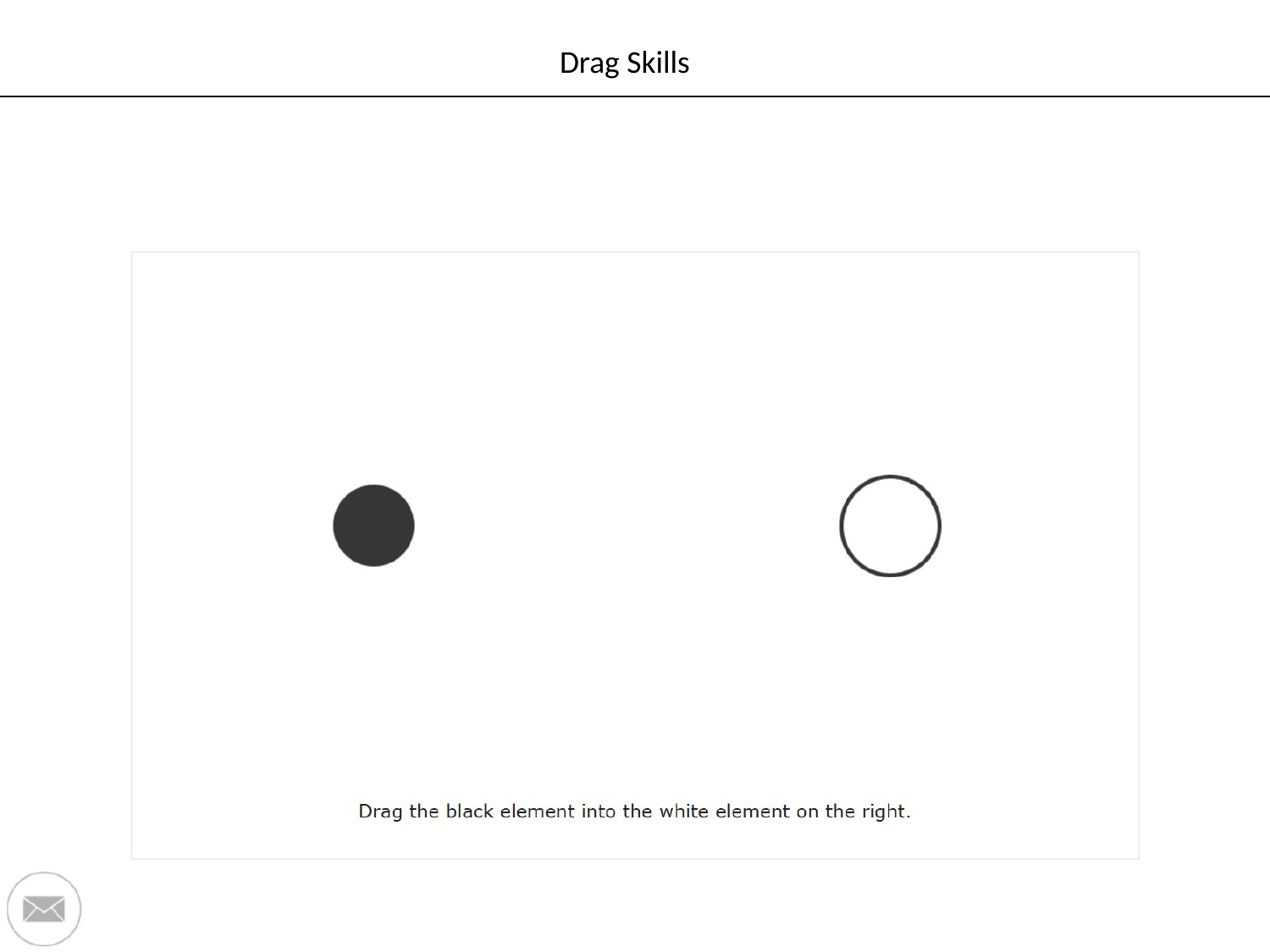

Drag Skills

## Slide 7
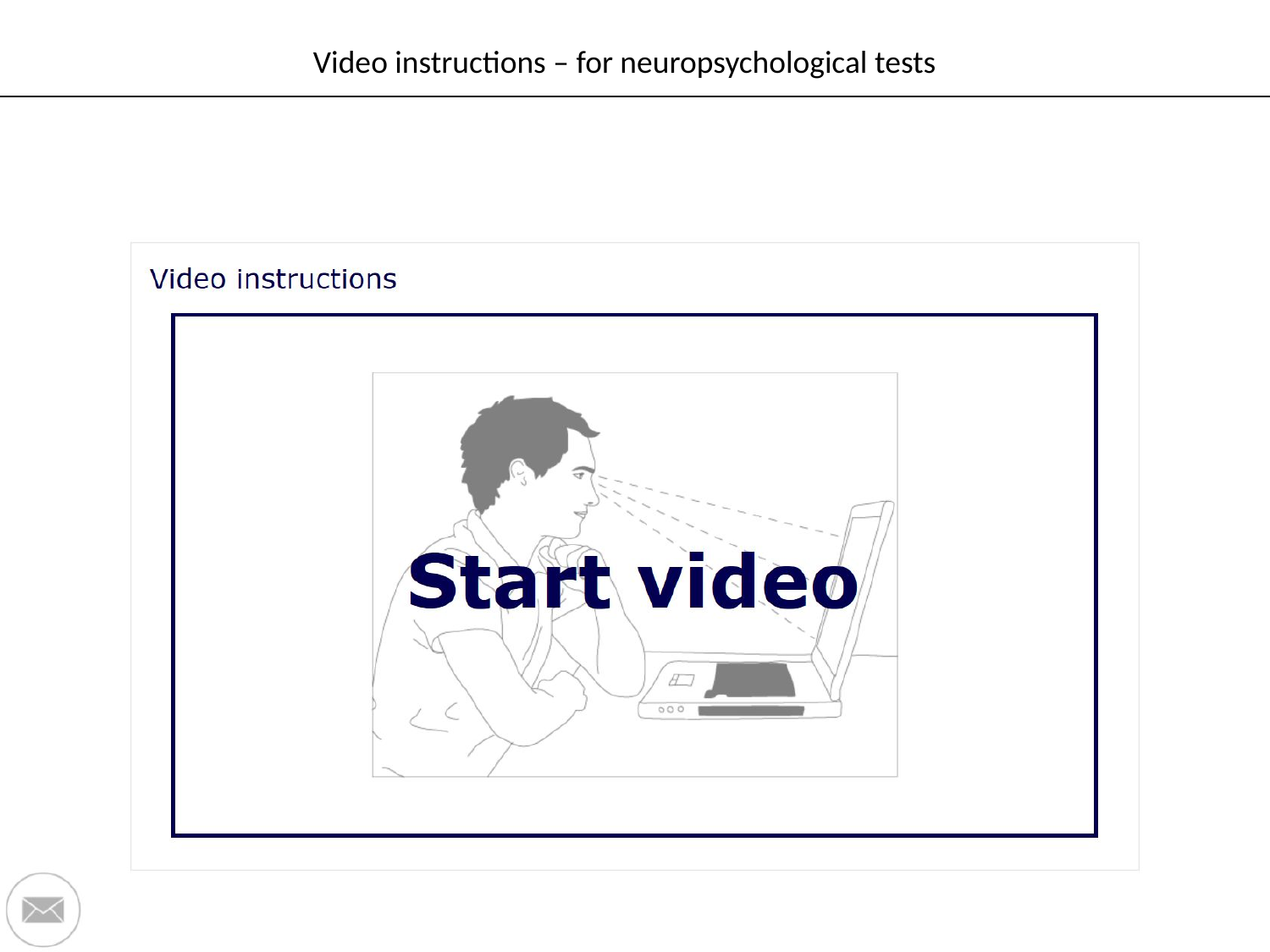

Video instructions – for neuropsychological tests

## Slide 8
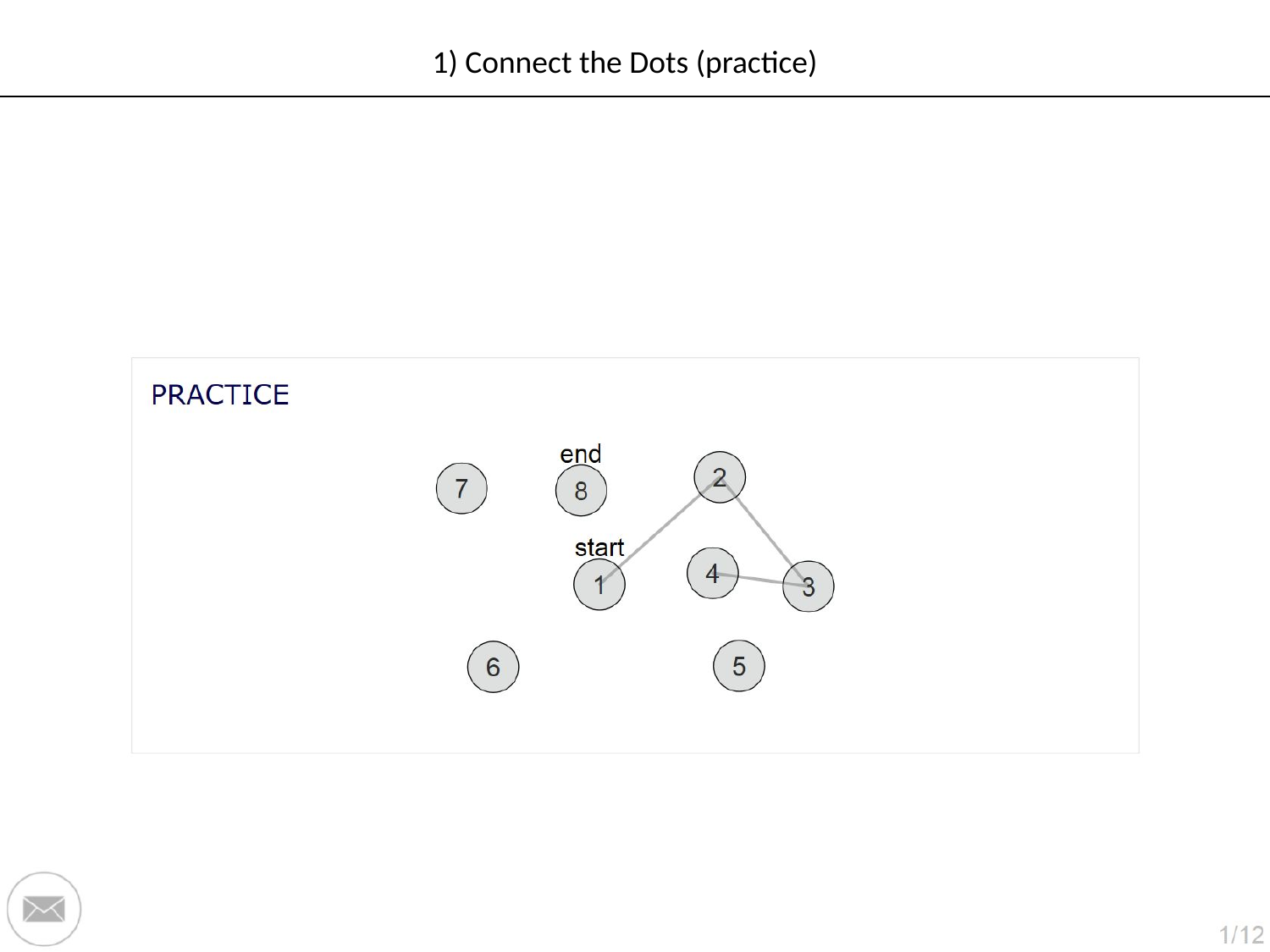

1) Connect the Dots (practice)

## Slide 9
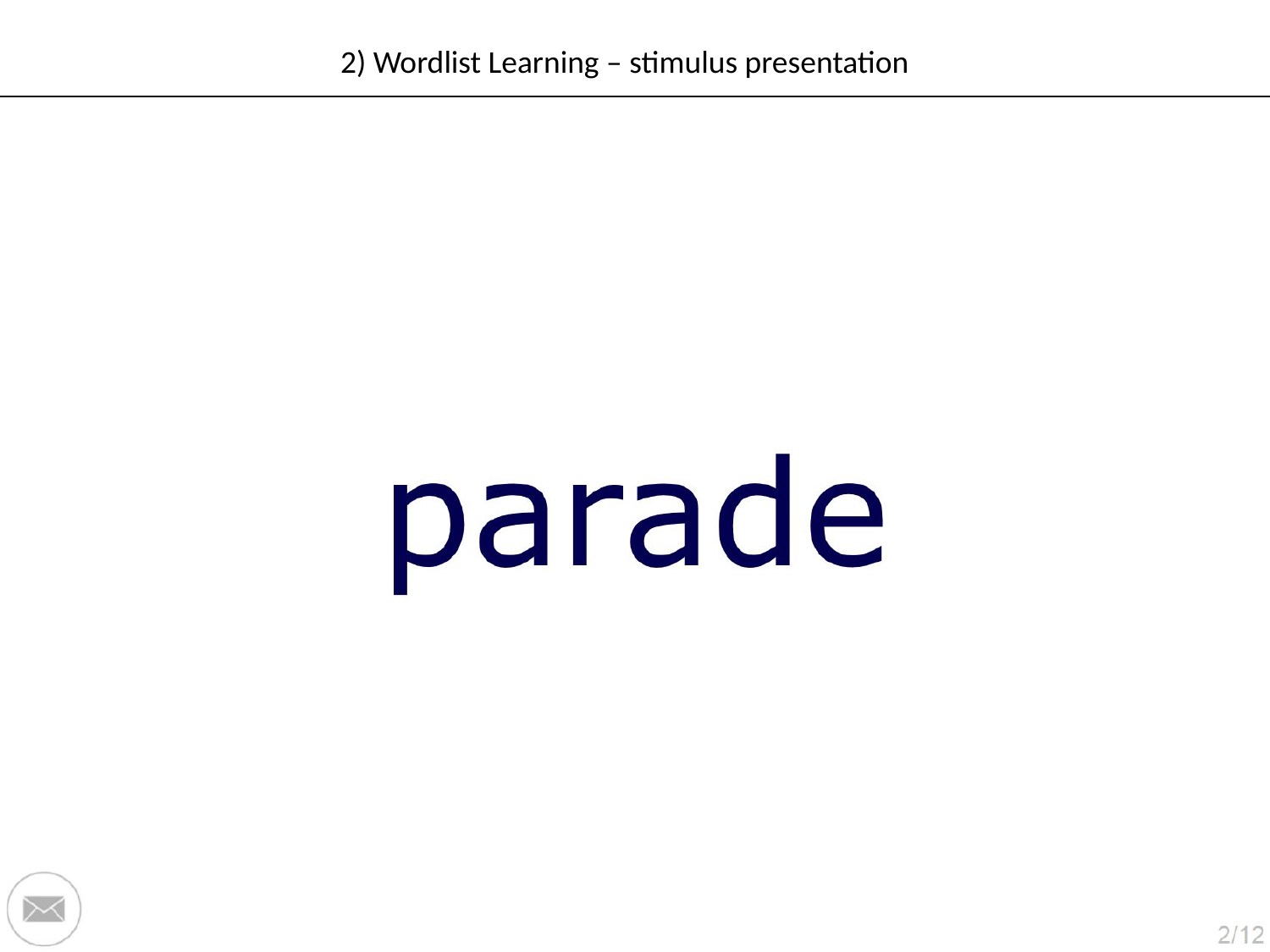

2) Wordlist Learning – stimulus presentation

## Slide 10
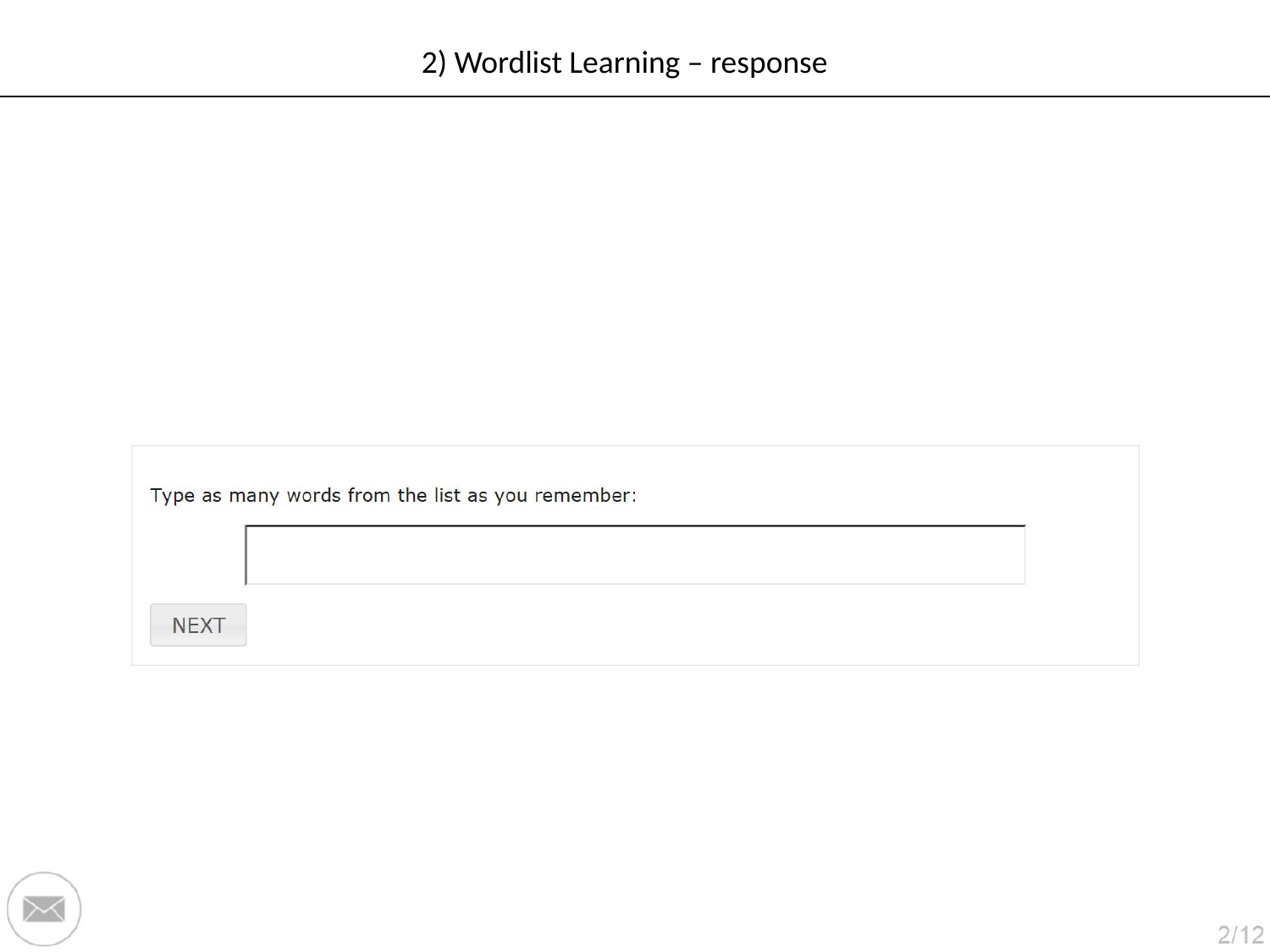

2) Wordlist Learning – response

## Slide 11
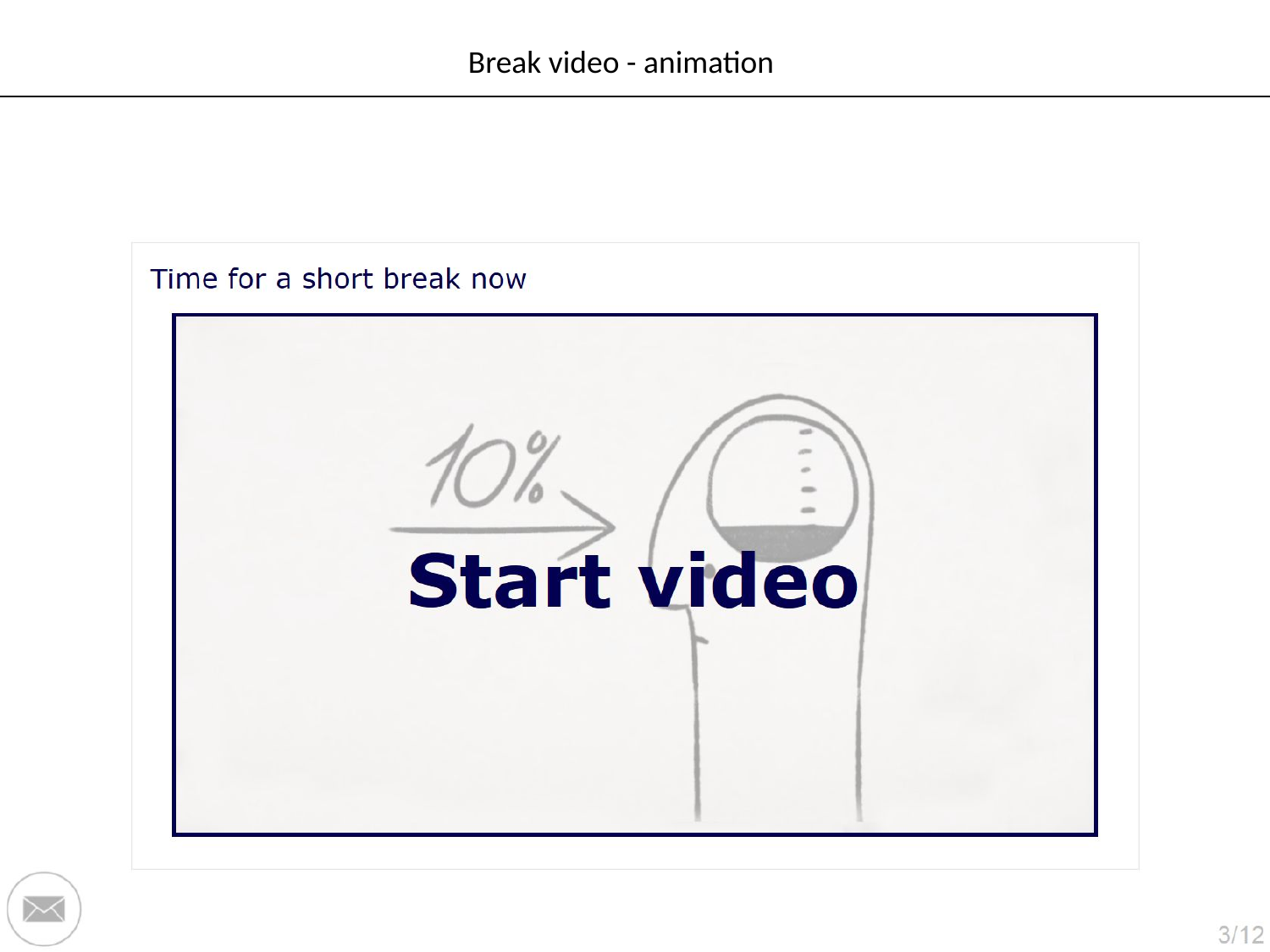

Break video - animation

## Slide 12
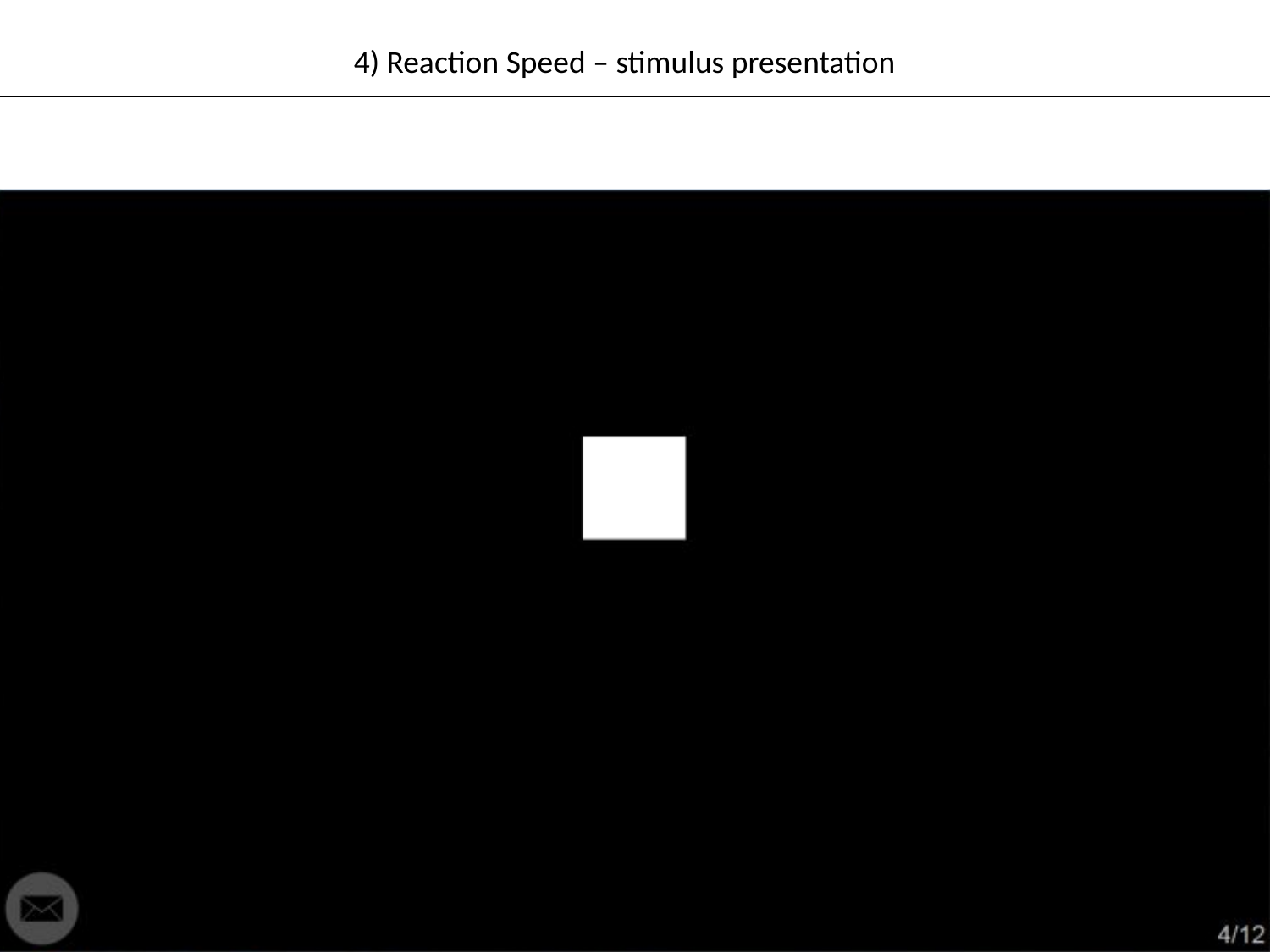

4) Reaction Speed – stimulus presentation

## Slide 13
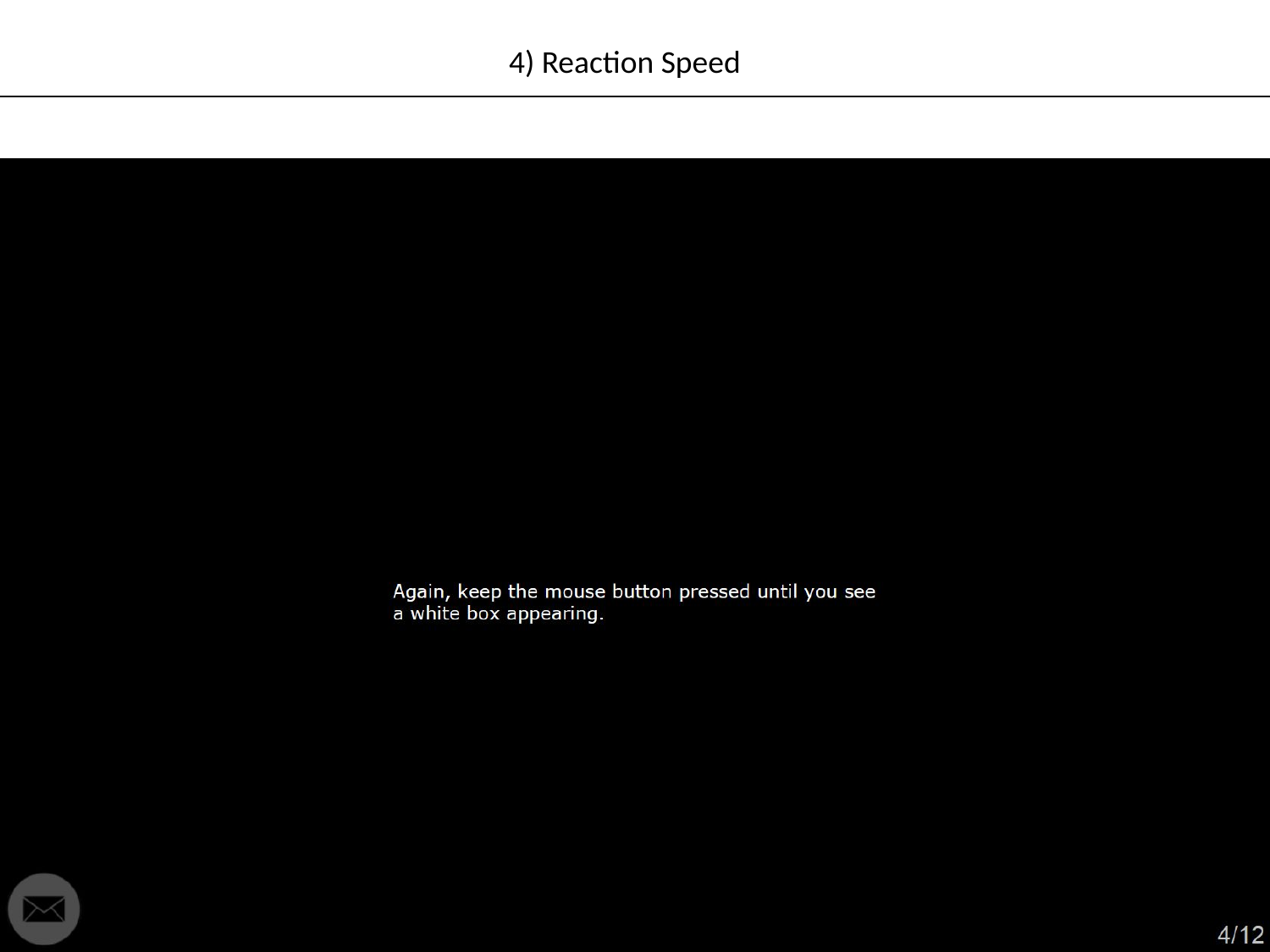

4) Reaction Speed

## Slide 14
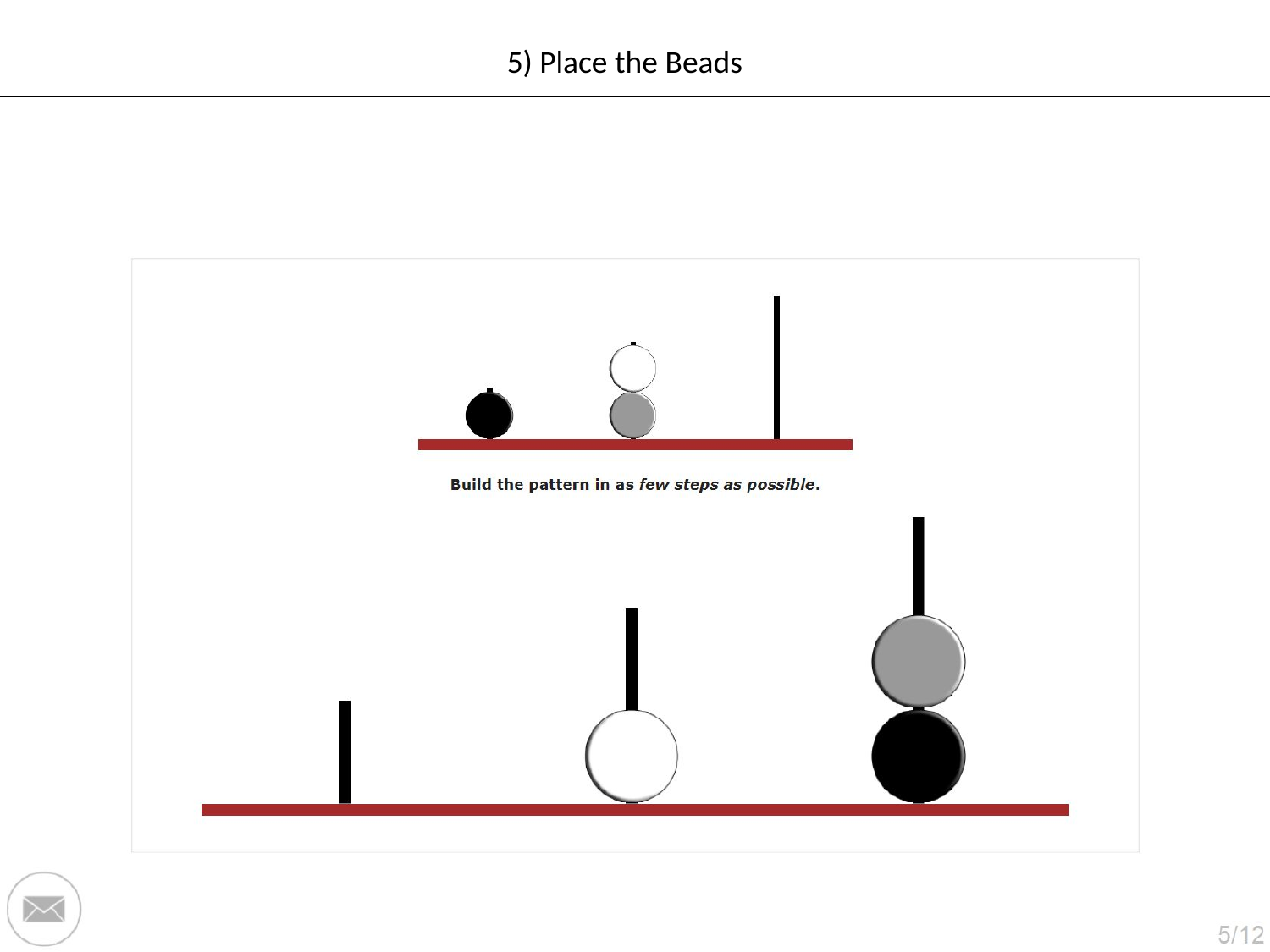

5) Place the Beads

## Slide 15
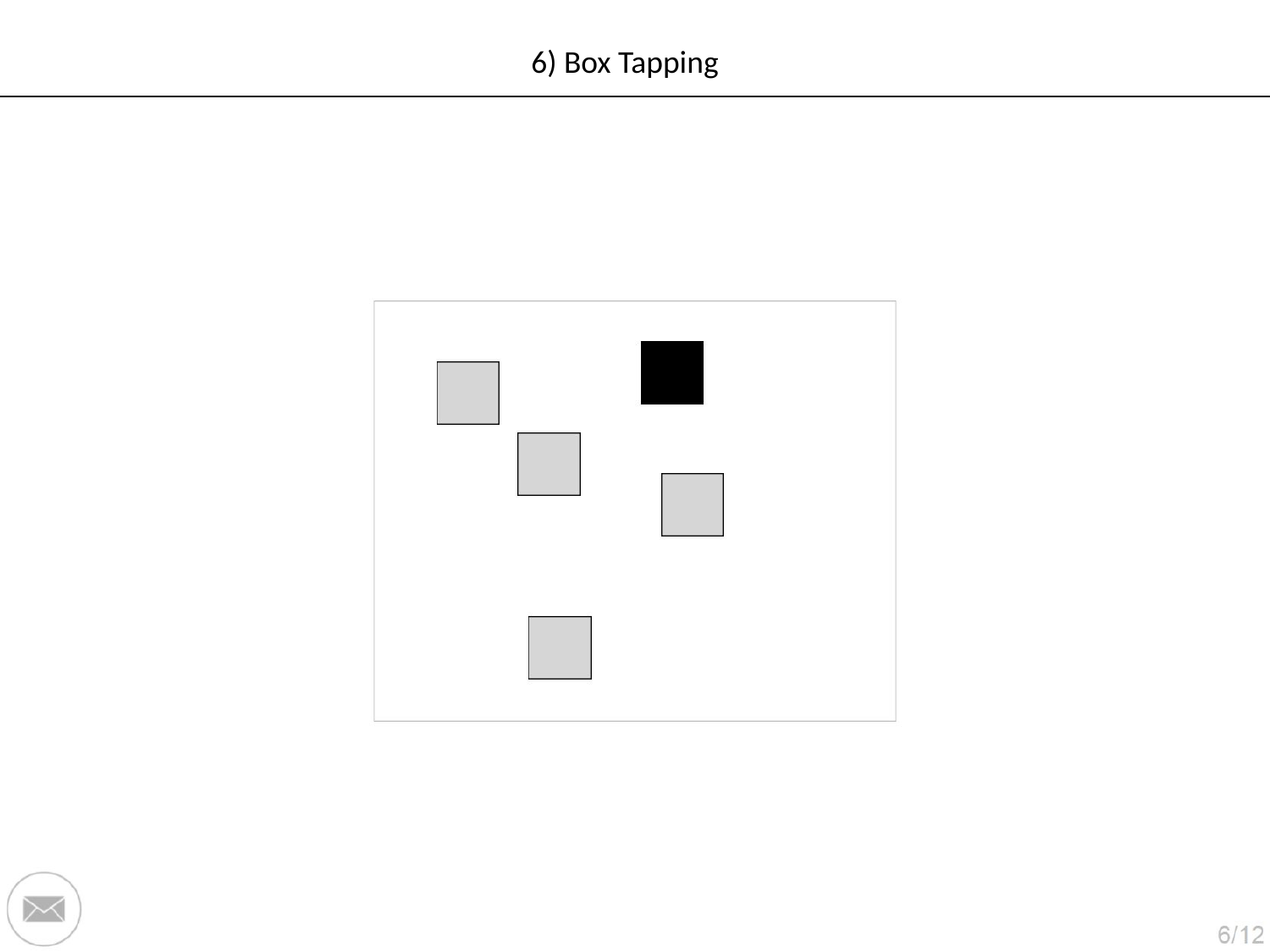

6) Box Tapping

## Slide 16
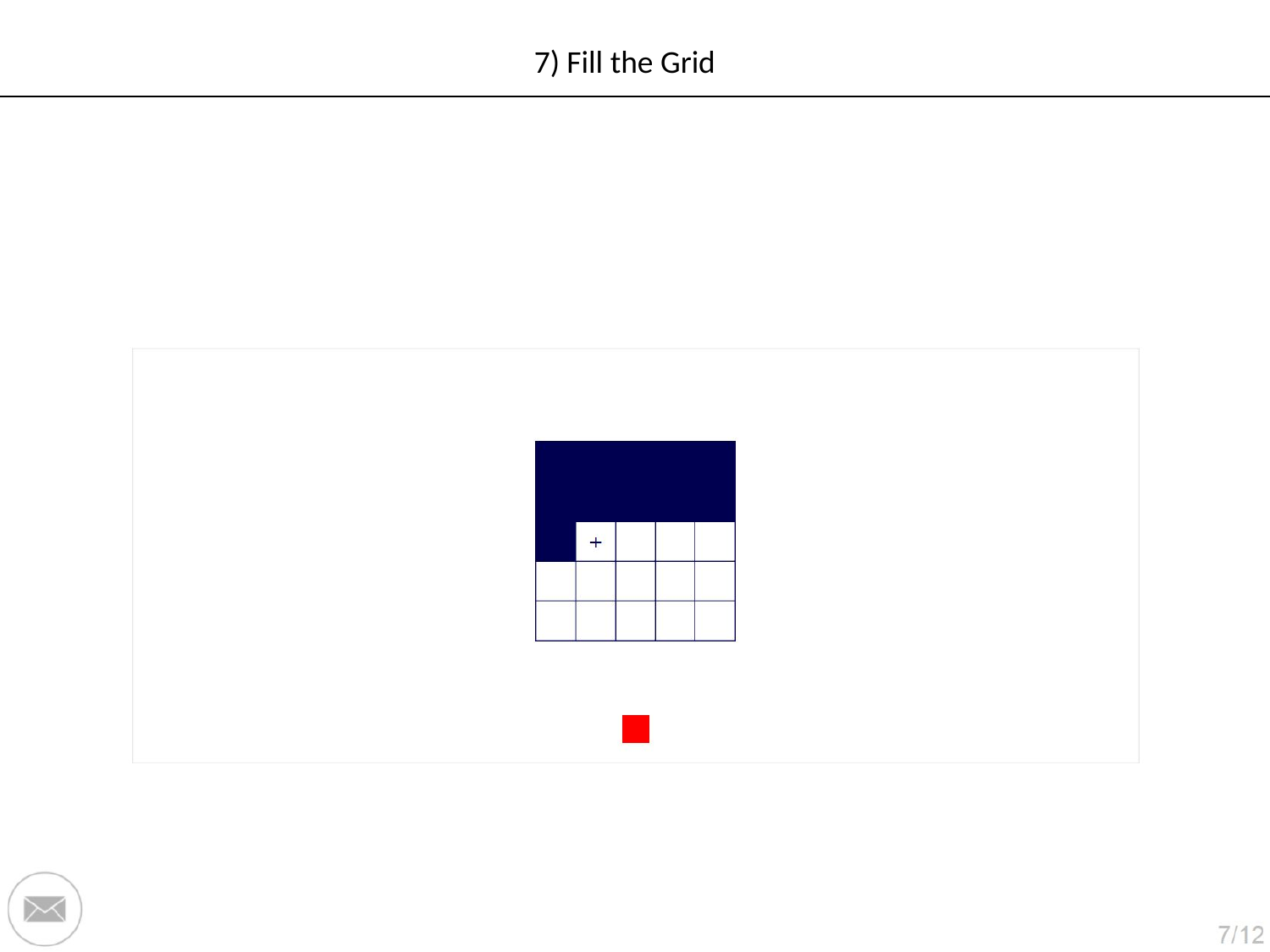

7) Fill the Grid

## Slide 17
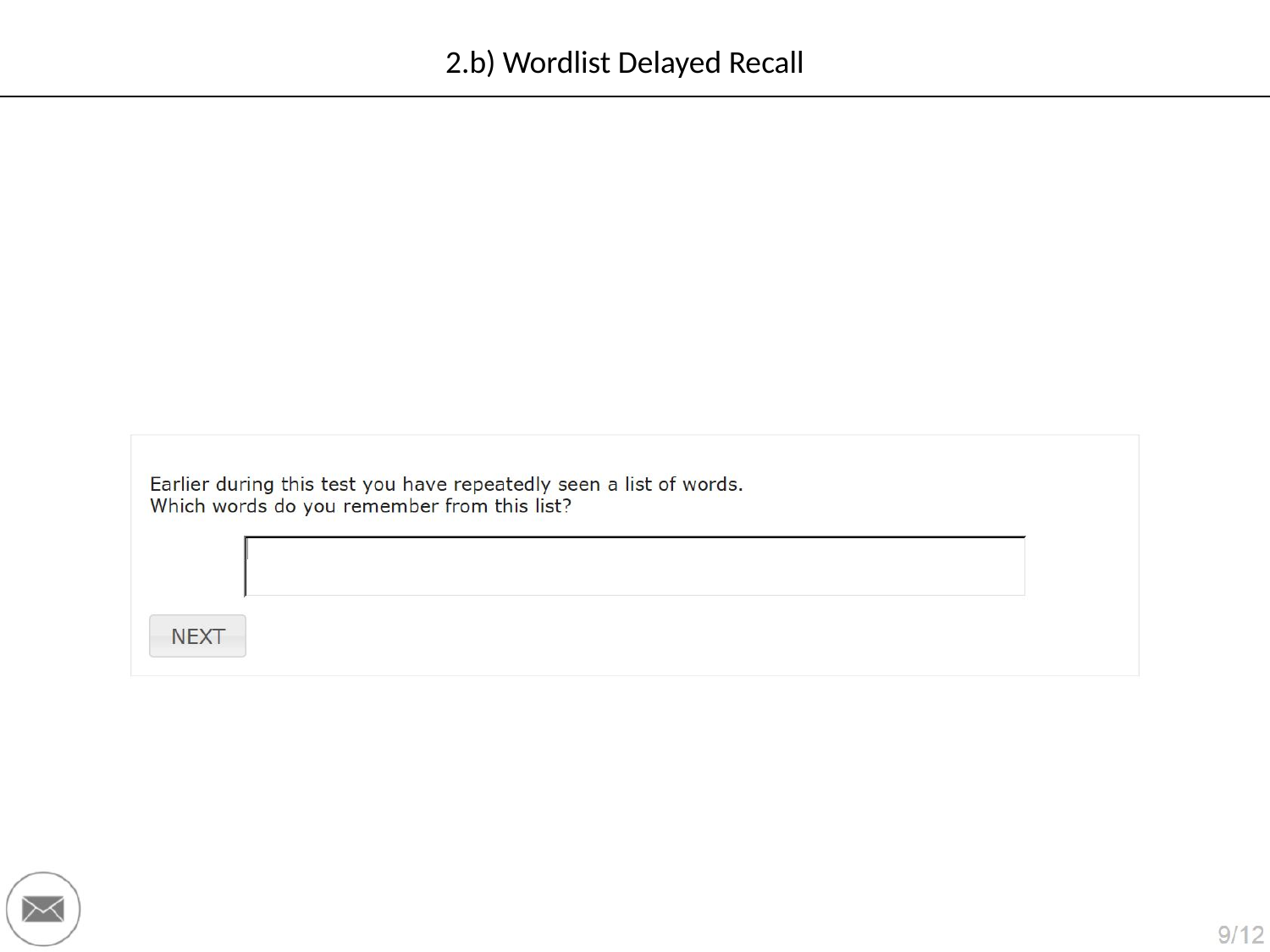

2.b) Wordlist Delayed Recall

## Slide 18
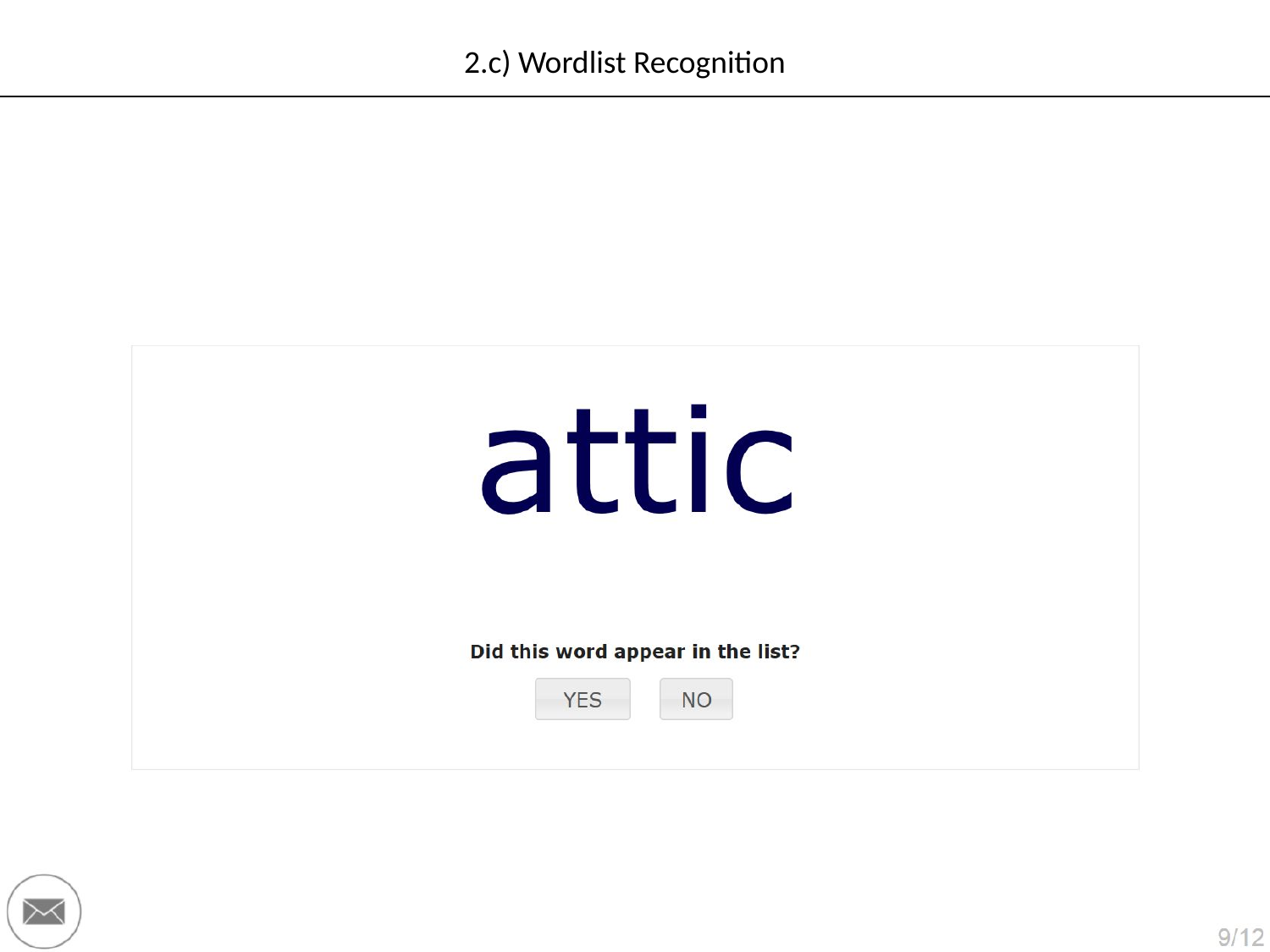

2.c) Wordlist Recognition

## Slide 19
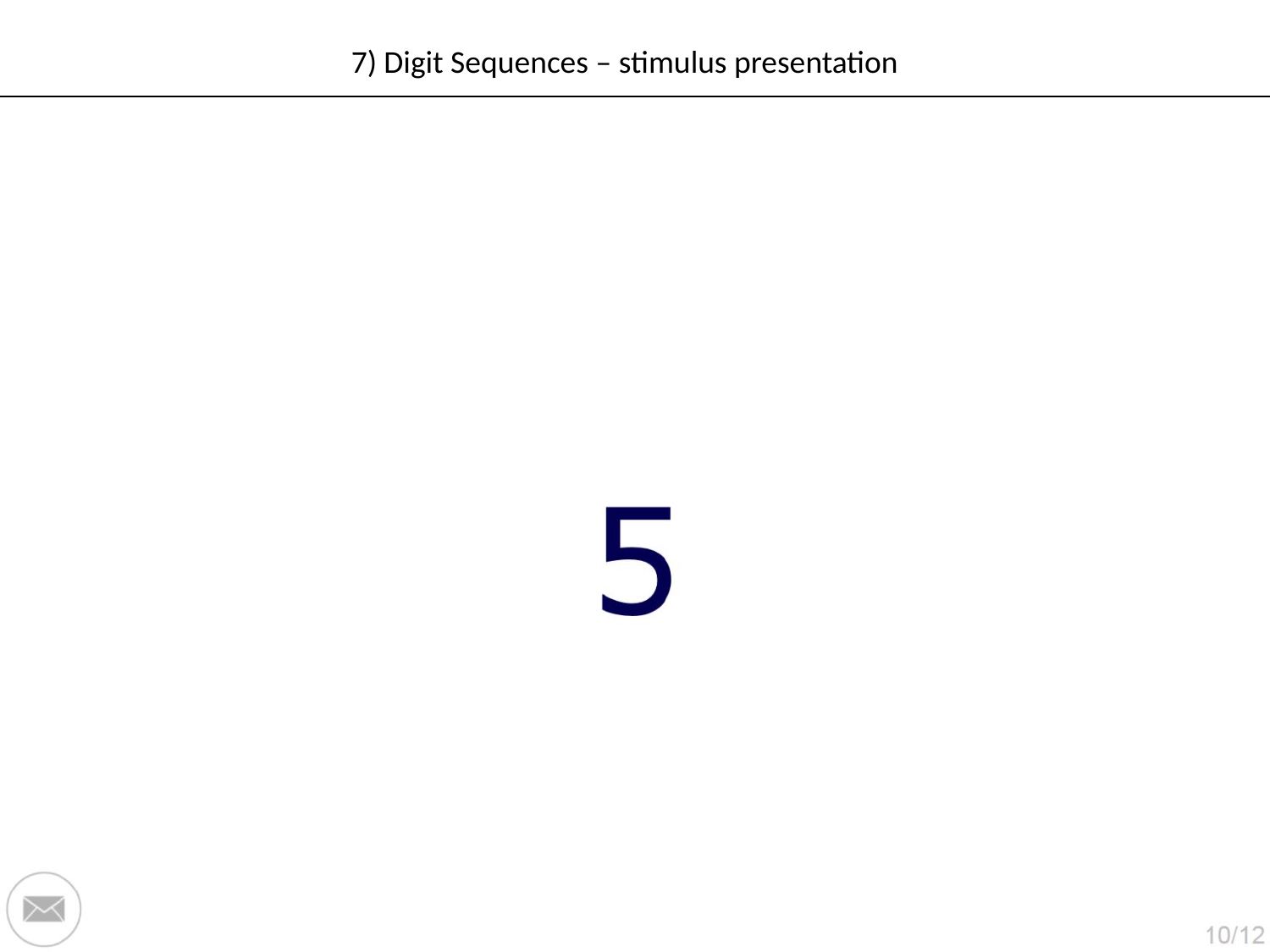

7) Digit Sequences – stimulus presentation

## Slide 20
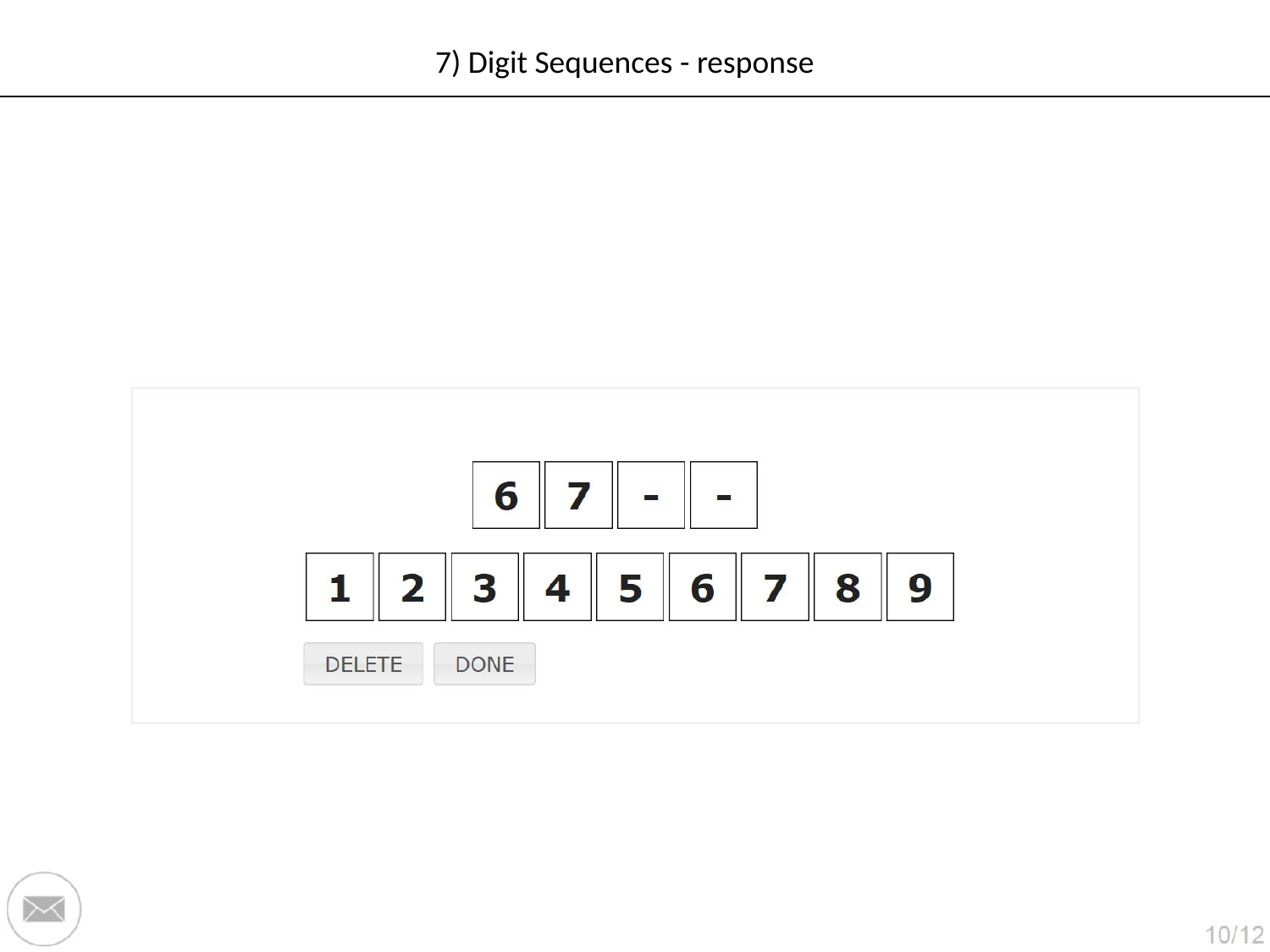

7) Digit Sequences - response
