# Supplementary material for: Online Self-Administered Cognitive Testing Using the Amsterdam Cognition Scan: Establishing Psychometric Properties and Normative Data
Source: J Med Internet Res. 2018 May 30;20(5):e192. doi: 10.2196/jmir.9298 (PMC6000479; doi:10.2196/jmir.9298)
Supplement: Multimedia Appendix 3 [file jmir_v20i5e192_app3.pdf]

Multimedia Appendix 3. Questionnaire results on test-retest reliability and practice effects (significant *t* values in italics). HADS: Hospital Anxiety and Depression Scale; ICC: intraclass correlation coefficient; MFI: Multidimensional Fatigue Inventory; SDC: smallest detectable change; SEM: standard error of measurement.

Questionnaire	n	Mean 1 (SD)	Mean 2 (SD)	*t* value (degrees of freedom), *P* value	SEM	SDC group[ab]	ICC[c]	Pearson *r*[c]	Spearman ρ[c]
HADS anxiety	248	5.09 (3.24)	4.75 (3.28)	*2.55*(247), *P*=.01	1.49	0.60	.79	.80	.78
HADS depression	248	3.11 (2.78)	3.02 (2.84)	0.69(247), *P*=.49	1.45	0.26	.74	.74	.72
MFI total	248	40.49 (15.27)	40.25 (14.99)	0.38(247), *P*=.70	7.02	1.24	.79	.79	.77

[a]SDC group: SDC / √n.

[b]For measures with a significant practice effect (*t* value in italics) Mean1 - Mean2 difference scores were added.

[c]All correlation coefficients were significant at *P*<.001.
