# Supplementary material for: Online Self-Administered Cognitive Testing Using the Amsterdam Cognition Scan: Establishing Psychometric Properties and Normative Data
Source: J Med Internet Res. 2018 May 30;20(5):e192. doi: 10.2196/jmir.9298 (PMC6000479; doi:10.2196/jmir.9298)
Supplement: Multimedia Appendix 4 [file jmir_v20i5e192_app4.pdf]

Multimedia Appendix 4. Multiple linear regression models including the factors age, age-squared, gender, education, and computer skills (tested) for Amsterdam Cognition Scan outcome measures.

All multiple regression analyses (MRA) are performed with normalized and standardized (mean 0, SD 1) scores. Education: 0=high, 1=low or medium; Gender: 0=female, 1=male.

Test	Variable	Beta	SE beta	Standard beta	*t* value	*P* value	R^2	SD (residual)
Connect the Dots I[ab]	Constant	.102	.074	–	1.38	–	–	–
	Age	.024	.004	.310	5.50	<.001	–	–
	Age-squared	.000	.000	.026	0.53,	.60	–	–
	Gender	-.257	.099	-.125	-2.59,	.01	–	–
	Education	-.099	.104	-.045	-0.96,	.34	–	–
	Computer skills	-.210	.026	-.453	-8.03,	<.001	.459	.724
Connect the Dots II[ac]	Constant	.099	.079	–	1.26	–	–	–
	Age	-.033	.005	-.422	-7.12,	<.001	–	–
	Age-squared	.000	.000	-.102	-2.03,	.04	–	–
	Gender	.077	.105	.038	0.74,	.46	–	–
	Education	-.142	.110	-.065	-1.29,	.20	–	–
	Computer skills	.136	.027	.294	4.96,	<.001	.392	.770
Wordlist Learning	Constant	.162	.095	–	1.71	–	–	–
	Age	-.028	.006	-.366	-5.13,	<.001	–	–
	Age-squared	.000	.000	-.057	-.93,	.35	–	–
	Gender	-.225	.127	-.109	-1.77,	.08	–	–
	Education	-.108	.132	-.049	-.82,	.42	–	–
	Computer skills	-.023	.033	-.051	-.70,	.48	.12	.930
Wordlist Delayed Recall	Constant	.160	.098	–	1.63	–	–	–
	Age	-.018	.006	-.237	-3.21,	.002	–	–
	Age-squared	.000	.000	-.049	-.78,	.44	–	–
	Gender	-.279	.132	-.135	-2.12,	.04	–	–
	Education	-.053	.137	-.024	-.39,	.70	–	–
	Computer skills	-.005	.034	-.011	-.15,	.88	.058	.962
Wordlist Recognition	Constant	.036	.098	–	.37	–	–	–
	Age	-.018	.006	-.229	-3.12,	.002	–	–
	Age-squared	.000	.000	-.074	-1.19,	.24	–	–
	Gender	-.117	.130	-.057	-.90,	.37	–	–
	Education	.241	.136	.110	1.77,	.08	–	–
	Computer	.032	.034	.070	.95,	.34	.068	.955

	skills							
Reaction Speed[ab]	Constant	.017	.098	–	.17	–	–	–
	Age	.012	.006	.159	2.16	P=.03	–	–
	Age-squared	0	0	.071	1.12	P=.26	–	–
	Gender	-.229	.129	-.112	-1.77	*P*=.08	–	–
	Education	.067	.135	.031	.49	*P*=.62	–	–
	Computer skills	-.085	.034	-.182	-2.47	*P*=.01	.087	.943
Box Tapping	Constant	-.015	.098	–	-.15	–	–	–
	Age	-.020	.006	-.258	-3.50	*P*=.001	–	–
	Age-squared	.000	.000	-.089	-1.42	*P*=.16	–	–
	Gender	.351	.130	.168	2.70	*P*=.007	–	–
	Education	-.172	.135	-.079	-1.28	*P*=.20	–	–
	Computer skills	.045	.034	.098	1.35	*P*=.18	.121	.930
Fill the Grid[ab]	Constant	.062	.076	–	.81	–	–	–
	Age	.021	.004	.271	4.78	*P*<.001	–	–
	Age-squared	.000	.000	.053	1.08	*P*=.28	–	–
	Gender	-.222	.101	-.108	-2.19	*P*=.03	–	–
	Education	-.005	.107	-.002	-.05	*P*=.96	–	–
	Computer skills	-.240	.027	-.489	-8.73	*P*<.001	.438	.741
Digit Sequences I	Constant	.058	.100	–	.59	–	–	–
	Age	-.013	.006	-.169	-2.27	*P*=.02	–	–
	Age-squared	.000	.000	-.022	-.35	*P*=.72	–	–
	Gender	.189	.133	.091	1.42	*P*=.16	–	–
	Education	-.364	.139	-.166	-2.62	*P*=.009	–	–
	Computer skills	-.032	.035	-.069	-.93	*P*=.36	.033	.976
Digit Sequences II	Constant	.045	.098	–	.46	–	–	–
	Age	-.018	.006	-.239	-3.24	*P*=.001	–	–
	Age-squared	.000	.000	-.027	-.43	*P*=.67	–	–
	Gender	.215	.130	.104	1.65	*P*=.10	–	–
	Education	-.327	.137	-.149	-2.39	*P*=.02	–	–
	Computer skills	-.017	.034	-.036	-.50	*P*=.62	.059	.956
Total score	Constant	.047	.048	–	.98	–	–	–
	Age	-.020	.003	-.445	-6.91	*P*<.001	–	–
	Age-squared	.000	.000	-.109	-1.93	*P*=.06	–	–
	Gender	.106	.063	.091	1.68	*P*=.09	–	–
	Education	-.136	.066	-.110	-2.06	*P*=.04	–	–
	Computer skills	.074	.017	.265	4.30	*P*<.001	.37	.44

[a]Reverse scoring was applied before MRA.

[b]Inverse transformations were applied (eg, 1/Connect the Dots I).

[c]Log10 transformation was applied.

[d]Squared root transformation was applied.
